# Revisão Sistemática sobre a Eficácia de Metas Intensivas do Tratamento Anti-Hipertensivo: Recomendação da Sociedade Brasileira de Cardiologia (SBC)

**DOI:** 10.36660/abc.20240761

**Published:** 2025-03-18

**Authors:** Andréa Araujo Brandão, Cibele Isaac Saad Rodrigues, Luiz Aparecido Bortolotto, Leonardo Castro Luna, Bruno Monteiro Barros, Mario Fritsch Toros Neves, Ana Flávia de Souza Moura, Frida Liane Plavnik, Luciano Ferreira Drager, Osni Moreira, Weimar Kunz Sebba Barroso de Souza, Wilson Nadruz

**Affiliations:** 1 Faculdade de Ciências Médicas Universidade do Estado do Rio de Janeiro Rio de Janeiro RJ Brasil Faculdade de Ciências Médicas – Universidade do Estado do Rio de Janeiro, Rio de Janeiro, RJ – Brasil; 2 Pontifícia Universidade Católica de São Paulo Sorocaba SP Brasil Pontifícia Universidade Católica de São Paulo, Sorocaba, SP – Brasil; 3 Instituto do Coração do Hospital das Clínicas Faculdade de Medicina Universidade de São Paulo São Paulo SP Brasil Instituto do Coração do Hospital das Clínicas da Faculdade de Medicina da Universidade de São Paulo, São Paulo, SP – Brasil; 4 Instituto Nacional de Cardiologia Rio de Janeiro RJ Brasil Instituto Nacional de Cardiologia, Rio de Janeiro, RJ – Brasil; 5 Escola Bahiana de Medicina e Saúde Pública Salvador BA Brasil Escola Bahiana de Medicina e Saúde Pública, Salvador, BA – Brasil; 6 Hospital Alemão Oswaldo Cruz São Paulo SP Brasil Hospital Alemão Oswaldo Cruz, São Paulo, SP – Brasil; 7 Clínica Gapski Moreira Curitiba PR Brasil Clínica Gapski Moreira, Curitiba, PR – Brasil; 8 Universidade Federal de Goiás Goiânia GO Brasil Universidade Federal de Goiás, Goiânia, GO – Brasil; 9 Faculdade de Ciências Médicas UNICAMP Campinas SP Brasil Faculdade de Ciências Médicas, Universidade Estadual de Campinas (UNICAMP), Campinas, SP – Brasil

**Keywords:** Hipertensão, Revisão Sistemática, Metanálise, Tratamento Farmacológico

## Abstract

**Fundamento:**

O controle rigoroso da pressão arterial tem sido investigado como uma estratégia para reduzir eventos cardiovasculares graves em pacientes hipertensos. No entanto, ainda existem dúvidas sobre o impacto de metas intensivas de tratamento anti-hipertensivo (meta < 130/80 mmHg) em comparação com metas convencionais (≥ 130/80 mmHg) na prevenção de infarto do miocárdio, acidente vascular cerebral, mortalidade e possíveis eventos adversos do tratamento.

**Objetivo:**

Avaliar a eficácia de metas intensivas de tratamento anti-hipertensivo na redução de eventos cardiovasculares críticos em comparação com metas usuais.

**Métodos:**

Esta revisão sistemática incluiu ensaios clínicos randomizados (ECRs) que compararam metas intensivas de controle da pressão arterial com metas convencionais em adultos com 18 anos ou mais. Foram incluídos estudos com pelo menos um dos seguintes desfechos: mortalidade, infarto do miocárdio, acidente vascular cerebral, progressão para doença renal crônica em estágios IV ou V, necessidade de diálise ou transplante renal. As bases de dados Medline, Embase e Cochrane Library foram pesquisadas até maio de 2024. A avaliação do risco de viés foi realizada por dois revisores independentes utilizando a ferramenta Risk of Bias 2 (RoB 2) da Colaboração Cochrane. A síntese dos resultados foi conduzida por meio de metanálise para o desfecho composto de infarto do miocárdio, acidente vascular cerebral e mortalidade por todas as causas. A certeza da evidência científica e a força de recomendação seguiram os métodos propostos pela ferramenta GRADE (Grading of Recommendations Assessment, Development and Evaluation).

**Resultados:**

Foram incluídos nove ECRs com mais de 34.000 participantes. O tratamento intensivo foi associado a uma redução de 13% nos eventos cardiovasculares. Nos estudos com baixo risco de viés, a redução foi de 17%, com alta certeza da evidência. Separadamente, observou-se uma redução significativa nos desfechos de infarto do miocárdio e acidente vascular cerebral, mas não na mortalidade por todas as causas. Dados limitados foram encontrados sobre a progressão da doença renal e necessidade de diálise ou transplante renal.

**Conclusão:**

As evidências de alta qualidade sugerem que metas mais intensivas de tratamento anti-hipertensivo reduzem significativamente os eventos cardiovasculares. Contudo, a escolha das metas de tratamento deve ser individualizada, considerando fatores como idade, fragilidade, o risco cardiovascular individual e a possibilidade de eventos adversos. A adesão ao tratamento é fundamental para o sucesso terapêutico.

## Graphical abstract

**Figura Central:**
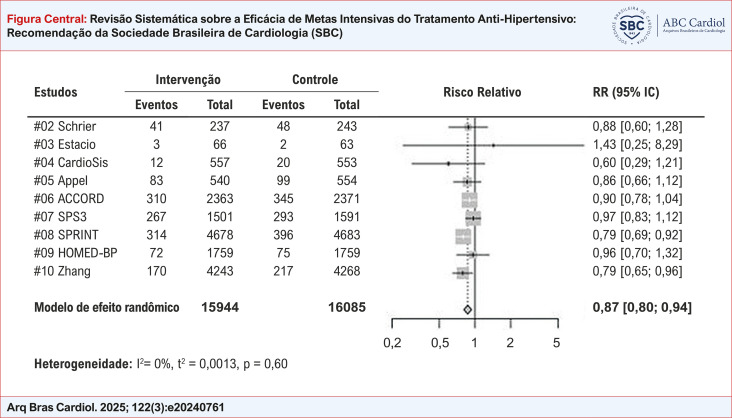
: Revisão Sistemática sobre a Eficácia de Metas Intensivas do Tratamento Anti-Hipertensivo: Recomendação da Sociedade Brasileira de Cardiologia (SBC)

**Figura Central:** : Revisão Sistemática sobre a Eficácia de Metas Intensivas do Tratamento Anti-Hipertensivo: Recomendação da Sociedade Brasileira de Cardiologia (SBC)

Heterogeneidade: I^2^= 0%, t^2^ = 0,0013, p = 0,60

**Recomendação 1:** A Sociedade Brasileira de Cardiologia recomenda a estratégia de metas mais intensivas para o tratamento anti-hipertensivo (valores menores de 130/80 mmHg) para pacientes adultos hipertensos, com o objetivo de reduzir eventos cardiovasculares importantes (infarto do miocárdio, acidente vascular cerebral e morte). Esta é uma recomendação forte com uma alta certeza da evidência.

**Recomendação 2:** A Sociedade Brasileira de Cardiologia recomenda a estratégia de metas mais intensivas para o tratamento anti-hipertensivo (valores menores de 130/80 mmHg) para pacientes hipertensos idosos (idade > 65 anos), com o objetivo de reduzir eventos cardiovasculares importantes futuros (infarto do miocárdio, acidente vascular cerebral e morte). Esta é uma recomendação forte com uma alta certeza da evidência. Esta recomendação deve ser avaliada de forma individualizada quando se tratar de um idoso frágil ou com expectativa de vida futura limitada.

**Recomendação 3:** A Sociedade Brasileira de Cardiologia recomenda a estratégia de metas mais intensivas para o tratamento anti-hipertensivo (redução adicional de 5 mmHg) para pacientes hipertensos de alto risco cardiovascular que já estejam dentro da meta de tratamento intensivo (PAS < 130 mmHg), com o objetivo de redução adicional de eventos cardiovasculares importantes futuros (infarto do miocárdio, acidente vascular cerebral e morte). Esta é uma recomendação forte com uma alta certeza da evidência.

## Introdução

A hipertensão arterial (HA) é atualmente um importante desafio de saúde, que contribui substancialmente para as taxas de doenças cardiovasculares globalmente. Trata-se de um dos principais fatores de risco para morbidade e mortalidade, afetando mais de um bilhão de pessoas e sendo responsável por aproximadamente 9,4 milhões de mortes anuais.^1^ A HA não apenas aumenta o risco de complicações cardiovasculares, mas também impõe uma considerável carga econômica sobre os sistemas de saúde, especialmente em países de baixa e média renda.^2^ No Brasil, a HA também é um grande problema de saúde pública, que afeta aproximadamente um terço da população adulta.^3^ É responsável por uma proporção significativa de infartos e acidentes vasculares cerebrais, que são as principais causas de morte no país.^4,5^ A HA também exerce uma influência substancial sobre o Sistema Único de Saúde (SUS), resultando em aumento dos custos de saúde, considerável perda de dias de trabalho e aposentadoria precoce.^6^ Há uma necessidade crescente de intervenções eficazes de saúde pública voltadas para a prevenção, detecção precoce e manejo da doença.^7^ Enfrentar esse desafio é crucial para melhorar a saúde e reduzir os níveis de doenças crônicas no Brasil.

O tratamento anti-hipertensivo desempenha papel crucial no manejo da doença e na redução do risco associado a eventos cardiovasculares, e normalmente envolve uma combinação de intervenções no estilo de vida com tratamentos farmacológicos.^7^ Atualmente, existe um debate a respeito das metas ideais a serem alcançadas na HA. As estratégias de controle intensivo, que visam metas mais baixas de pressão arterial (PA), mostraram uma redução significativa na incidência de eventos críticos em pacientes de alto risco em estudos clínicos como o SPRINT.^8^ No entanto, essa abordagem também traz um aumento potencial dos riscos de eventos adversos, como hipotensão, lesão renal aguda e desequilíbrios eletrolíticos, que podem levar a desfechos desfavoráveis, especialmente em pacientes idosos ou frágeis.^9^ Por outro lado, o tratamento convencional, que segue metas de PA mais moderadas, pode não proporcionar o mesmo nível de proteção cardiovascular e renal.^8^ Essa discussão ressalta a importância de tratamentos individualizados, que considerem o estado geral de saúde do paciente, além das comorbidades e a possibilidade de efeitos adversos relacionados ao tratamento.

Com o objetivo de esclarecer a melhor estratégia em relação às metas de PA em pacientes hipertensos, considerando as melhores evidências científicas atuais, a Sociedade Brasileira de Cardiologia (SBC) desenvolveu uma Recomendação Clínica sobre o tema.

## Métodos

Para a realização da Recomendação Clínica, foi realizada uma revisão sistemática. A pergunta de pesquisa estruturada no formato PICO (paciente/população, intervenção, comparação e desfecho) foi: Em pacientes hipertensos, qual é a diferença do tratamento farmacológico com metas mais intensivas de controle da PA (< 130/80 mmHg) comparadas a metas de controle habitual (≥ 130/80 mmHg), especificamente em relação a desfechos críticos e importantes, como morte, acidente vascular cerebral, infarto do miocárdio, progressão para insuficiência renal estágio 4 ou 5, necessidade de diálise ou transplante renal? O protocolo do estudo foi registrado na base de dados PROSPERO, sob o número CRD42024545853.

A revisão sistemática rápida, metodologia empregada neste documento, pertence à família das revisões sistemáticas. Trata-se de uma ferramenta desenvolvida na última década com o objetivo de manter o rigor metodológico em busca das melhores evidências possíveis, porém com modificações que aceleram o tempo de execução. Geralmente, essas revisões informam sociedades médicas ou instituições de saúde sobre as melhores evidências disponíveis, com base em uma pergunta no formato PICO, de maneira sensível, transparente e sistemática. Instituições de destaque na área de metodologia descreveram os métodos para esse tipo de revisão sistemática.^10-12^

Uma primeira busca por revisões sistemáticas sobre a pergunta PICO foi realizada em três bases de dados: Medline, Embase e a Biblioteca Cochrane. Os detalhes das estratégias de busca estão fornecidos no material suplementar (Tabela S1). Dois pesquisadores realizaram, de forma independente, a seleção dos estudos e a extração das características basais dos documentos definidos para leitura completa (Tabelas S2 e S3). No entanto, entre as revisões sistemáticas encontradas, que cobrem os anos de 2019 a 2024, nenhuma abordou exatamente a questão PICO de interesse, exceto pelo documento de Sakima et al.^13^ Esta publicação incluiu buscas por estudos até março de 2018, recuperando nove artigos que compararam a meta intensiva de PA (< 130/80 mmHg) com a meta usual e avaliaram eventos cardiovasculares como desfecho. A decisão dos autores foi atualizar a busca dessa revisão sistemática e realizar uma nova metanálise com os dados extraídos dos estudos primários.

Uma segunda busca por ensaios clínicos randomizados (ECRs) foi realizada para a atualização do documento de referência escolhido, englobando o período de março de 2018 a maio de 2024 nas mesmas bases de dados que a primeira busca. Os detalhes das estratégias de busca estão fornecidos no material suplementar (Tabela 4S). Os artigos selecionados para a revisão de texto completo foram considerados para inclusão se atendessem aos seguintes critérios: (1) ser um ECR; (2) apresentar estimativas de risco para avaliar o impacto de metas intensivas de PA versus metas usuais de PA; (3) apresentar pelo menos um dos seguintes desfechos descrito numericamente: mortalidade por todas as causas, acidente vascular cerebral, infarto do miocárdio e progressão para insuficiência renal estágio 4 ou 5, necessidade de diálise ou transplante renal; (4) incluir pacientes com 18 anos ou mais. Não houve restrições de idioma na seleção dos estudos.

Dois pesquisadores foram responsáveis pela seleção, extração e avaliação da qualidade de todos os artigos finais. A ferramenta RoB 2 (*Risk of Bias 2*) da Colaboração Cochrane foi utilizada para a avaliação do risco de viés.^14^ A força das recomendações e a certeza das evidências foram determinadas de acordo com a metodologia GRADE (*Grading of Recommendations Assessment, Development and Evaluation*)^15^ (Tabelas 2 e 3).Uma descrição detalhada da metodologia empregada nesta revisão sistemática pode ser encontrada no material suplementar. O viés de publicação não foi avaliado pela razão do número de estudos originais ser menor do que dez.

As recomendações clínicas foram definidas de forma consensual em reunião de um Painel de Recomendação formado por profissionais indicados pela SBC. Foi considerado o limiar de 5% como uma diferença mínima importante para a intervenção ser considerada clinicamente relevante.

**Tabela 2:** – Risco de viés dos estudos primários segundo a ferramenta RoB 2.0 da Colaboração Cochrane

Estudo	Intervenção	Comparador	Desfecho	D1	D2	D3	D4	D5	
Schrier	TTO Intensivo	TTO Usual	Eventos CV	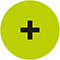	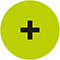	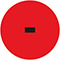	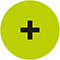	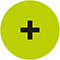	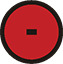
ESTACIO	TTO Intensivo	TTO Usual	Eventos CV	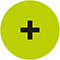	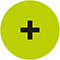	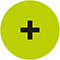	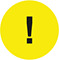	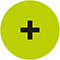	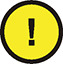
CardioSis	TTO Intensivo	TTO Usual	Eventos CV	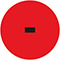	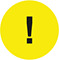	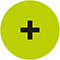	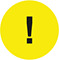	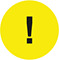	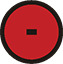
Appel	TTO Intensivo	TTO Usual	Eventos CV	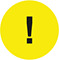	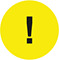	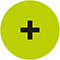	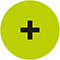	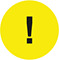	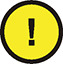
ACCORD	TTO Intensivo	TTO Usual	Eventos CV	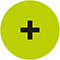	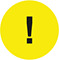	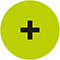	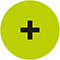	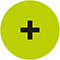	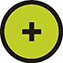
SPS3	TTO Intensivo	TTO Usual	Eventos CV	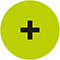	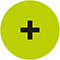	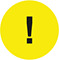	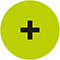	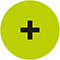	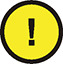
SPRINT	TTO Intensivo	TTO Usual	Eventos CV	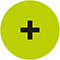	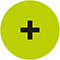	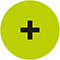	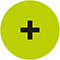	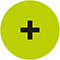	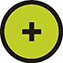
HOMED-BP	TTO Intensivo	TTO Usual	Eventos CV	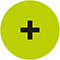	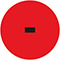	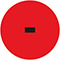	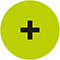	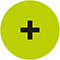	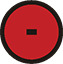
STEP - Zhang	TTO Intensivo	TTO Usual	Eventos CV	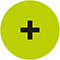	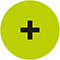	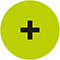	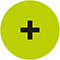	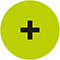	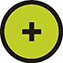

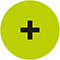
: Baixo Risco; 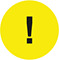: Moderado Risco; 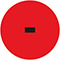: Alto Risco. D1: Randomização; D2: Desvio do TTO; D3: Dados Faltantes; D4: Medida do Desfecho; D5: Seleção de Resultados Reportados. CV: cardiovasculares; TTO: tratamento.

**Tabela 3:** – Sumários de resultados (SoF – Summary of Findings), de acordo com a metodologia GRADE

Desfecho	Efeito Absoluto (95% CI)		Efeito Relativo (95% IC)	N^o^ de participantes (estudos)	Certeza da Evidência
	Risco com o tratamento usual	Risco com o tratamento intensivo (< 130/80 mmHg)			
Eventos CV (Morte, IAM e AVC)	89 por 1000	77 por 1000	RR 0,87 (0,80 a 0,94)	32749 (9 ECRs)	Alta
Morte	39 por 1000	36 por 1000	RR 0,93 (0,80 a 1,09)	32029 (9 ECRs)	Alta

AVC: acidente vascular cerebral; CV: cardiovasculares; ECRs: ensaios clínicos randomizados; IAM: infarto agudo do miocárdio; Nº: número.

Todo o projeto foi supervisionado e financiado pela SBC. A elaboração da revisão sistemática foi conduzida por uma equipe independente de metodologistas.

## Resultados

Os nove artigos incluídos na metanálise de referência,^13^ comparando a meta de tratamento anti-hipertensivo intensivo (< 130/80 mmHg) com a meta de tratamento padrão, foram avaliados para inclusão nesta nova metanálise aqui apresentada. Desses, um foi excluído por não apresentar os riscos relativos de interesse,^16^ e os oito restantes foram incluídos.^8,17-23^

Como resultado da segunda busca na literatura, que teve como objetivo identificar novos ECRs publicados após março de 2018, foram inicialmente triadas 2.061 citações. Destas, apenas dois estudos foram selecionados para a revisão de texto completo, resultando na inclusão de um ECR adicional na metanálise atualizada,^24^ e na exclusão do segundo artigo por não ser um ECR original.^25^

As principais características dos nove estudos primários incluídos nesta metanálise estão apresentadas na Tabela 1. Todas as características extraídas estão disponíveis no material suplementar (Tabela 5S).

**Tabela 1:** – Características principais dos estudos primários

Autor principal	Ano de Publicação	Características da População	Meta da PA no grupo Intensivo	PA Sistólica Basal (mmHg)	PA Diastólica Basal (mmHg)	Número de participantes	Eventos	Idade Média	Percentual de Mulheres	País	Dados Faltantes n(%)
#02 Schrier	2002	Pacientes com diabetes tipo 2 normotensos	<130/80 mmHg	141,5	96	480	89	57	49%	EUA	55(11)
#3 Estacio	2006	Pacientes normotensos com diabetes tipo 2 e normo ou microalbuminúria	< 75 mmHg (PAD)	126	84	129	5	56	47%	EUA	10(7)
#4 CardioSis	2009	Pacientes hipertensos com síndrome metabólica	<130 mmHg	144,0	92,0	1111	97	55,6	43%	Itália	27(2)
#5 Appel	2010	Pacientes afro-americanos com nefroesclerose hipertensiva	<130/80 mmHg	142	95	1094	120	55,3	39%	EUA	0(0)
#6 ACCORD	2010	Pacientes com diabetes tipo 2 e hipertensão	<120 mmHg	139,3	76,0	4733	460	62,2	38%	EUA	232(4,9)
#7 SPS3	2013	Pacientes com acidente vascular cerebral lacunar recente	<130 mmHg	143,4	78,2	3020	377	63	37%	EUA	550(18,4)
#8 SPRINT	2015	Pacientes hipertensos com risco cardiovascular aumentado	<120 mmHg	139,7	78,1	9361	562	67,9	36%	EUA	986(10,5)
#9 HOMED-BP	2018	Pacientes japoneses com hipertensão, maiores de 40 anos	<125/80	154	90	3518	51	59,6	50%	Japão	710(20)
#10 Zhang	2021	Pacientes chineses com idade entre 60 e 80 anos com hipertensão	110-130 mmHg	146,1	86,1	8511	355	66,2	54%	China	234(2,7)
**Autor principal**	**Ano de Publicação**	**Desfechos Primários**	**Eventos CV Grupo Intensivo**	**Eventos CV Grupo Usual**	**Morte Grupo Intensivo**	**Morte Grupo Usual**	**IAM Grupo Intensivo**	**IAM Grupo Usual**	**AVC Grupo Intensivo**	**AVC Grupo Usual**	**RoB 2.0**
#02 Schrier	2002	Alteração no clearance de creatinina	41/237	48/243	18/237	20/243	19/237	15/243	4/237	13/243	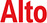
#3 Estacio	2006	Alteração na excreção urinária de albumina	3/66	2/63	1/66	0/63	Não relatado	Não relatado	Não relatado	Não relatado	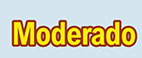
#4 CardioSis	2009	Redução no índice de massa ventricular esquerda	12/557	20/553	4/557	5/553	4/557	6/553	4/557	9/553	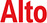
#5 Appel	2010	Desfecho composto de morte, doença renal em estágio terminal ou redução da taxa de filtração glomerular	83/540	99/554	38/540	47/554	19/540	23/554	26/540	29/554	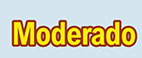
#6 ACCORD	2010	Desfecho composto de IAM, AVC e morte	310/2363	345/2371	150/2363	144/2371	126/2363	146/2371	34/2363	55/2371	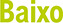
#7 SPS3	2013	AVC recorrente	267/1501	293/1519	106/1501	101/1519	36/1501	40/1519	125/1501	152/1519	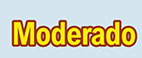
#8 SPRINT	2015	Desfecho composto de IAM, AVC, morte cardiovascular, SCA e IC	314/4678	396/4683	155/4678	210/4683	97/4678	116/4683	62/4678	70/4683	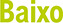
#9 HOMED-BP	2018	Desfecho composto de morte cardiovascular, IAM e AVC	72/1759	75/1759	27/1759	31/1759	25/1759 (***)	28/1759 (***)	20/1759	16/1759	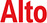
#10 Zhang	2021	Desfecho composto de eventos cardiovasculares e morte	170/4243	217/4268	67/4243	64/4268	55/4243 (**)	82/4268 (**)	48/4243	71/4268	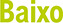

(**) SCA, não IAM isolado; (***) Não apenas IAM, a doença isquêmica do coração abrangeu morte por angina pectoris, parada cadíaca e infarto do miocárdio não fatal. AVC: acidente vascular cerebral; CV: cardiovasculares; IAM: infarto agudo do miocárdio; IC: insuficiência cardíaca; PA: pressão arterial; PAD: pressão arterial diastólica; SCA: síndrome coronariana aguda.

**Figura 1:**
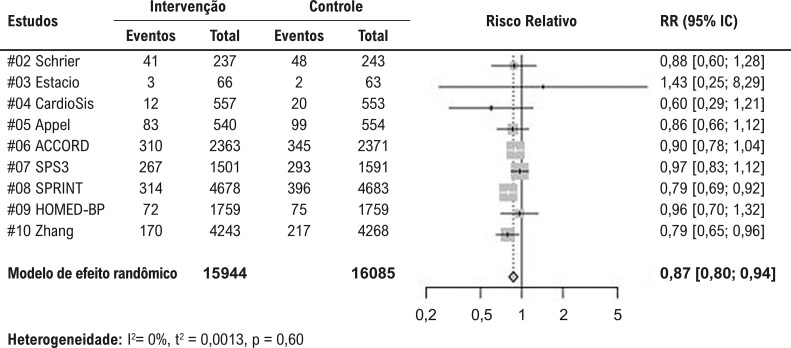
– Metanálise para o desfecho composto primário, definido como infarto do miocárdio, acidente vascular cerebral e mortalidade por todas as causas.

**Figura 2:**
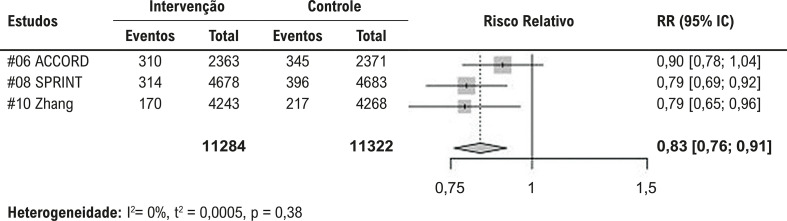
– Metanálise para o desfecho composto primário, definido como infarto do miocárdio, acidente vascular cerebral e mortalidade por todas as causas, avaliado apenas para os estudos com baixo risco de viés.

O efeito para o desfecho composto primário, obtido pela sumarização dos dados dos nove estudos primários, é apresentado na Figura 1. Houve uma redução de 13% nos eventos, favorecendo a estratégia de metas mais intensivas de tratamento anti-hipertensivo. Para o mesmo desfecho, quando apenas estudos com baixo risco de viés foram incluídos (Figura 2), observou-se uma redução de 17% nos eventos, também favorecendo a estratégia de metas mais intensivas de tratamento anti-hipertensivo. A certeza das evidências para essa análise foi classificada como alta, de acordo com a metodologia GRADE.

Heterogeneidade: I^2^= 0%, t^2^ = 0,0013, p = 0,60

Heterogeneidade: I^2^= 0%, t^2^ = 0,0005, p = 0,38

Quando avaliados separadamente, esses três desfechos mostraram uma redução no infarto do miocárdio e no acidente vascular cerebral, favorecendo a estratégia de metas mais intensivas, sem diferença significativa observada para a mortalidade por todas as causas (Figura 1S).

O desfecho de progressão da doença renal foi avaliado em quatro estudos incluídos, mas apenas dois forneceram dados numéricos. Devido ao baixo número de estudos e às diferenças nas definições de progressão renal, os autores não incluíram esse desfecho na avaliação primária e não realizaram uma metanálise para esse evento individual.

Duas revisões sistemáticas adicionais avaliadas para leitura completa na primeira busca respondem perguntas PICO com pequenas diferenças em relação à pergunta científica original desta revisão. Ambas foram consideradas pelos autores como temas muito relevantes para o sistema de saúde e subsidiaram duas recomendações clínicas adicionais deste documento.^26,27^ Os dois documentos foram considerados de boa qualidade técnica pelos autores, tendo sido penalizados pela avaliação de qualidade AMSTAR 2^28^ apenas em características que não comprometiam a informação central. A avaliação de qualidade pela ferramenta AMSTAR 2 destas duas revisões sistemáticas adicionais utilizadas para recomendações clínicas suplementares estão na Tabela 6S.

A Tabela 7S descreve o PRISMA deste documento.

## Discussão

A HA é um dos principais fatores de risco modificável para morte prematura. Portanto, determinar se metas mais intensivas de redução da PA oferecem benefícios adicionais para a redução de complicações cardiovasculares é de suma importância, alinhando-se à missão educativa da SBC. Esta revisão oferece uma resposta conclusiva para essa lacuna na prática clínica.

Definir o valor-alvo para o tratamento anti-hipertensivo tem sido um desafio na literatura científica devido à variabilidade das metas propostas em diferentes estudos. Embora um ponto de corte de 120/80 mmHg possa ser considerado, ECRs que visaram esse alvo mais rigoroso, frequentemente enfrentaram dificuldades em atingir esses resultados. Por essa razão, nesta revisão sistemática, optou-se por incluir estudos nos quais o braço de intervenção visava uma PA abaixo de 130/80 mmHg, objetivo mais alcançável na prática clínica real.

Na busca inicial da literatura, que teve como objetivo identificar revisões sistemáticas que abordassem a pergunta PICO predefinida, nenhum documento foi encontrado que pudesse fornecer uma resposta atualizada. Por isso, decidiu-se atualizar o artigo de Sakima et al.^13^

Com base nos resultados demonstrados nesta revisão, que inclui dados de mais de 34.000 indivíduos, o benefício clínico de metas mais rigorosas de controle da PA para a população de pessoas com HA está bem estabelecido. No entanto, ainda havia incerteza se todos os espectros de risco cardiovascular se beneficiariam desse efeito. Entre os nove estudos incluídos, cinco — com mais de 6.000 pacientes — tinham uma idade média inferior a 60 anos e uma proporção significativa de mulheres (40% ou mais). Isso nos permite concluir que uma parte substancial desses participantes estava na categoria de baixo risco de eventos cardiovasculares (risco estimado < 7,5% para 10 anos). Por outro lado, os quatro estudos restantes, que incluíam pacientes em sua maioria homens com mais de 60 anos, representavam adequadamente o outro extremo de exposição, abrangendo pacientes com alto risco cardiovascular (risco estimado > 15% para 10 anos). Combinando esses dados, podemos supor que estes subgrupos estavam bem representados nos estudos que observaram um benefício importante em redução de eventos.

Outra pergunta relevante que se coloca é se reduções adicionais para valores de 120/80 mmHg ou ainda mais baixos, particularmente em pacientes com maior risco cardiovascular e menor probabilidade de efeitos colaterais, poderiam resultar em benefícios clínicos adicionais (e valeria recomendar, se colocados em prática). Com base na metanálise de dados individuais da *Blood Pressure Lowering Treatment Trialists’ Collaboration* de 2021, para qualquer faixa inicial de PA (mesmo em indivíduos com valores sistólicos menores do que 120 mmHg), uma redução de 5 mmHg determina uma redução de 11% nos eventos cardiovasculares.^27^ Para esses indivíduos de alto risco, buscar as metas de controle rigoroso propostas pelos estudos SPRINT^8^ e ACCORD^21^ pode ser uma opção terapêutica vantajosa. Deve-se levar em consideração o risco de efeitos colaterais, número de medicamentos utilizados (adesão) e custos adicionais, contrapondo-se com a significativa redução de eventos cardiovasculares críticos. Esta revisão sistemática serviu de referência para a recomendação clínica de número três neste documento.

Metas mais rigorosas de controle anti-hipertensivo sempre levantam preocupações sobre eventos adversos, especialmente em pacientes muito idosos ou frágeis. No entanto, uma metanálise recente envolvendo 20.895 indivíduos idosos demonstrou que a estratégia de metas intensivas de controle da PA resultou em uma redução de 29% nos principais eventos cardiovasculares, sem evidências de aumento de reações adversas graves ou piora da função renal.^26^ Esses achados reforçam ainda mais a ideia de implementar um controle mais rigoroso da HA para a população em geral, independentemente da idade. No entanto, essa recomendação não elimina a necessidade de se considerar as circunstâncias individuais de cada paciente e de se estabelecer metas menos intensivas para aqueles que são frágeis, têm expectativa de vida limitada ou possuem características que possam aumentar o risco de efeitos colaterais. Esta revisão sistemática serviu de referência para a recomendação clínica de número dois neste documento.

Ainda sobre possíveis eventos adversos do tratamento anti-hipertensivo, existe a discussão sobre o fenômeno da curva J. Este se refere à observação de que tanto níveis excessivamente altos quanto excessivamente baixos de PA estão associados a um aumento do risco de complicações cardiovasculares, formando uma curva em forma de “J” quando plotada em um gráfico. Esse fenômeno é particularmente observado com a PA diastólica e sua associação com a doença arterial coronariana e outros eventos cardiovasculares. O conceito da relação em curva J entre a PA e desfechos cardiovasculares foi proposto pela primeira vez na década de 1970,^29^ e posteriormente verificado em vários estudos observacionais, como por exemplo o Estudo de Framingham.^30^ Baseado na mesma revisão sistemática realizada pelo grupo *Blood Pressure Lowering Treatment Trialists’ Collaboration* de 2021, reunindo 48 ECRs (que possuem uma certeza da evidência mais robusta do que estudos observacionais), quando avaliamos a população com doença cardiovascular prévia e reduções da PA sistólica além de 120 mmHg, não foi evidenciado um aumento de eventos cardiovasculares. Ao contrário, mesmo nesta população de muito alto risco e metas muito intensivas de redução da PA, foi observada também a diminuição dos desfechos primários, contradizendo a hipótese do fenômeno da curva J.

Uma discussão relevante adicional é se o método de medida da PA poderia influenciar os resultados de efeito clínico observados. O estudo SPRINT utilizou um método que envolvia a realização de três aferições de PA durante uma visita ao consultório, com o paciente sentado, após um período de cinco minutos de repouso, utilizando um sistema de medida automatizada (Modelo 907, Omron Healthcare), sem a presença do médico ou outro profissional de saúde. Este procedimento foi criticado por diferir daqueles utilizados em outros estudos, levantando preocupações sobre a reprodutibilidade de seus resultados. No entanto, um estudo conduzido logo após o SPRINT demonstrou, por meio de entrevistas com os seus participantes, que essa técnica foi implementada em menos da metade dos pacientes, sem diferenças nos desfechos clínicos entre os grupos com base no método de mensuração da PA.^31^ Um segundo estudo comparou a técnica proposta no ensaio SPRINT com medidas de PA feitas sem repouso prévio. Os resultados mostraram uma variabilidade considerável na concordância entre os valores, sugerindo que a técnica do SPRINT poderia levar a leituras de PA mais baixas.^32^ No entanto, este foi um estudo observacional, conduzido em um único centro, e com um pequeno número de participantes. Essas críticas são importantes, mas não invalidam os resultados apresentados nesta metanálise. Destaca-se assim, uma das muitas vantagens a respeito da realização de uma revisão sistemática, que sumariza os resultados de vários estudos, minimizando a influência de um único artigo isolado no resultado final. Ao realizar uma análise de sensibilidade excluindo o estudo SPRINT, o benefício clínico da redução intensiva da PA permanece, mostrando uma redução de 11% nos eventos (Figura 2S).

Recente revisão sistemática em processo de publicação avaliou pergunta PICO semelhante e chegou a resultados parecidos, corroborando as recomendações clínicas sugeridas neste documento da SBC.^33^ Whelton et al.^33^ observaram para a meta intensiva de controle da PA sistólica (< 130 mmhg) uma redução de 22% para um desfecho composto de acidente vascular cerebral, doença coronariana, insuficiência cardíaca e morte cardiovascular (*hazard ratio* [HR] = 0,78; intervalo de confiança [IC] de 95% =0,70 a 0,87; heterogeneidade: I^2^ = 64,5%; p = 0,01). Houve diferença estatística para o desfecho mortalidade geral (HR = 0,89; IC 95% =0,79 a 0,99; heterogeneidade: I^2^ = 37%; p = 0,14), embora haja algum grau de imprecisão para a importância clínica deste efeito, comprovado pelo IC.

Finalmente, um ECR publicado já na fase final de escrita deste documento também corrobora os achados desta revisão sistemática.^34^ Liu et al.^34^ incluíram 11.255 participantes chineses de alto risco cardiovascular (4.359 com diabetes e 3.022 com acidente vascular cerebral prévio) que foram designados para tratamento intensivo (n = 5.624) ou tratamento padrão (n = 5.631). A idade média foi de 64,6 anos. A média da PA sistólica durante o acompanhamento foi de 119,1 mmHg (desvio padrão: 11,1) no grupo de tratamento intensivo e 134,8 mmHg (desvio padrão: 10,5) no grupo de tratamento padrão. Durante uma mediana de 3,4 anos de acompanhamento, desfechos primários ocorreram em 547 (9,7%) participantes no grupo de tratamento intensivo e 623 (11,1%) no grupo de tratamento padrão (HR = 0,88; IC 95% = 0,78 a 0,99). Eventos adversos graves de síncope foram infrequentes, e ocorreram com maior frequência no grupo de tratamento intensivo (n = 24 [0,4%] de 5.624) do que no grupo de tratamento padrão (n = 8 [0,1%] de 5.631; HR = 3,00, IC 95% = 1,35 a 6,68). Não houve diferença significativa entre os grupos nos eventos adversos graves de hipotensão, anormalidade eletrolítica, queda prejudicial ou injúria renal aguda.

Outros fatores, além de benefícios e malefícios clínicos, devem ser considerados por um Painel de Recomendações ao tomar a decisão final para uma recomendação clínica, de acordo com a estrutura de Evidências para Decisão da metodologia GRADE. O custo é um desses fatores. No Brasil, as principais classes de medicamentos anti-hipertensivos são fornecidas gratuitamente pelo Ministério da Saúde em todo o país, o que facilita a implementação da estratégia intensiva recomendada neste documento.^35^

A adesão dos pacientes aos medicamentos prescritos pode ser um desafio, pois metas mais rigorosas de controle da PA normalmente exigem o uso maior de medicamentos para alcançar os resultados desejados. Estratégias para melhorar a adesão, como simplificar os esquemas posológicos (prescrevendo medicamentos que requerem um menor número de doses por dia),^36^ utilizar medicamentos combinados em um único comprimido,^37^ incentivar os pacientes a monitorarem sua PA no domicílio^38^ e promover uma relação próxima entre os profissionais de saúde e os pacientes,^39^ podem contribuir para o sucesso da estratégia.

Outra abordagem para melhorar a adesão dos pacientes é envolvê-los no processo de tomada de decisão. Utilizando calculadoras de predição de risco cardiovascular, é possível determinar a probabilidade individual de eventos cardiovasculares nos próximos 10 anos.^40^ Com base nessa estimativa, os pacientes podem ser esclarecidos sobre o real significado de uma redução adicional na PA e consequentemente no risco cardiovascular futuro, considerando também as possíveis desvantagens (eventos adversos, custo, adesão, etc.). Isso permite uma melhor compreensão da estratégia proposta como um todo, e o efeito da estratégia por tempo indeterminado.

Esta revisão sistemática e metanálise apresenta algumas limitações. Primeiramente, há uma compreensível heterogeneidade entre as características dos diversos estudos sobre o tema. Eles diferem em vários aspectos, incluindo as definições das metas de tratamento intensivo anti-hipertensivo, a idade média dos participantes, os métodos de medida da PA e o risco cardiovascular basal dos pacientes incluídos, entre outros. Em segundo lugar, há informações limitadas nos estudos em relação à progressão da doença renal em pacientes já acometidos por doença renal crônica. Embora este tenha sido um desfecho clínico predefinido no protocolo, não foi amplamente investigado nos estudos incluídos, o que impede a formulação de conclusões definitivas. Por último, o tempo de seguimento dos estudos variou entre dois e cinco anos, aproximadamente. O tempo de tratamento para HA é indefinido, a princípio para o total de vida restante do paciente hipertenso. Portanto, o efeito clínico comprovado em um número limitado de anos pode estar subdimensionado para uma estratégia clínica de muito mais longo prazo.

## Conclusão

A HA continua sendo um enorme desafio em saúde, com profundas implicações para a morbidade e mortalidade cardiovascular da população brasileira. Os achados desta revisão ressaltam os benefícios das metas intensivas de tratamento anti-hipertensivo, que demonstraram reduzir significativamente a incidência de eventos cardiovasculares importantes. No entanto, a decisão de adotar metas mais agressivas de controle da PA deve ser individualizada, levando-se em consideração fatores específicos do paciente, como idade, fragilidade e risco de eventos adversos. Além disso, garantir a adesão dos pacientes aos esquemas medicamentosos prescritos é crucial para o sucesso de qualquer estratégia de tratamento. Ao considerar os potenciais benefícios e riscos, envolvendo o paciente na tomada de decisão final, os profissionais de saúde podem otimizar o manejo da HA e melhorar os resultados clínicos do paciente e do sistema de saúde.
