# Supplementary material for: Revisão Sistemática sobre a Eficácia de Metas Intensivas do Tratamento Anti-Hipertensivo: Recomendação da Sociedade Brasileira de Cardiologia (SBC)
Source: Arq Bras Cardiol. 2025 Mar 18;122(3):e20240761. [Article in Portuguese] doi: 10.36660/abc.20240761 (PMC12013737; doi:10.36660/abc.20240761)
Supplement: Supplementary file 1 [file 0066-782X-abc-122-3-e20240761-suppl01.pdf]

MATERIAL SUPLEMENTAR

Revisão sistemática sobre a eficácia de metas intensivas de tratamento anti-hipertensivo - Recomendação da Sociedade Brasileira de Cardiologia (SBC).

Tabela 1S - 1a busca por revisões sistemáticas.

Tabela 2S - características basais da revisões sistemáticas recuperadas na primeira busca nas bases de dados.

Tabela 3S – referências das revisões sistemáticas listadas na tabela 2S.

Tabela 4S - 1a busca por artigos originais.

Tabela 5S – características dos artigos originais

Tabela 6S – AMSTAR 2 das revisões sistemáticas adicionais utilizadas para recomendações clínicas adicionais.

Tabela 7S - PRISMA da revisão sistemática.

Figura 1S – Metanálises dos desfechos individuais.

Figura 2S – Metanálise dos desfechos compostos sem o estudo SPRINT.

8. Metodologia detalhada

Tabela 1S - 1a busca por revisões sistemáticas (data 05/07/2024).

**PUBMED**
(("hypertension"[MeSH Terms] OR "blood pressure"[MeSH Terms] OR "hypertension"[Title/Abstract] OR "blood pressure"[Title/Abstract] OR "blood pressure high"[Title/Abstract] OR "high blood pressure*"[Title/Abstract]) AND "intensive"[Title/Abstract]) AND ((systematicreview[Filter]) AND (2014:2024[pdat]))
**EMBASE**

('hypertension'/exp OR 'htn (hypertension)' OR 'acute hypertension' OR 'arterial hypertension' OR 'blood pressure, high' OR 'cardiovascular hypertension' OR 'controlled hypertension' OR 'endocrine hypertension' OR 'high blood pressure' OR 'high renin hypertension' OR 'hypertension' OR 'hypertensive disease' OR 'hypertensive effect' OR 'hypertensive response' OR 'neurogenic hypertension' OR 'preexistent hypertension' OR 'salt high blood pressure' OR 'salt hypertension' OR 'secondary hypertension' OR 'systemic hypertension') AND ('antihypertensive agent'/exp OR 'anti hypertensic agent' OR 'anti hypertensive' OR 'anti hypertensive agent' OR 'anti hypertensive drug' OR 'antihypertension agent' OR 'antihypertensive' OR 'antihypertensive agent' OR 'antihypertensive agents' OR 'antihypertensive drug' OR 'antihypertensives' OR 'antihypertonic agent' OR 'hypotensiva' OR 'hypotensive' OR 'hypotensive agent' OR 'hypotensive drug') AND intensive AND ('meta analysis'/de OR 'systematic review'/de)
**COCHRANE**
#1 MeSH descriptor: [Hypertension] explode all trees<br>#2 hypertension OR "high blood pressure" OR "blood pressure, high"<br>#3 #1 OR #2<br>#4 intensive<br>#5 #3 AND #4<br>Filters: Topics + Heart & circulation + Hypertension + Date (01/01/2014 to 05/07/2024)

Tabela 2S - características basais da revisões sistemáticas recuperadas na primeira busca nas bases de dados

Estudo	ANO	1o AUTOR	OBS	Bases	Estudos	POPULAÇÃO	Definição Intensivo
#1	2023	Seidu	População de Idosos (PICO diferente)	4	6	≥ 65 anos (DM e não-DM)	(< 120 a <140) x (< 140 a < 160)
#2	2023	Yan	População de Idosos (PICO diferente)	3	6	≥ 60 anos	(<130) x (130-139) x (>140)
#3	2023	Triatmaja	Artigo não recuperado	4	7	≥ 60 anos	Não disponível
#4	2022	Chen	População de Idosos (PICO diferente)	1	6	≥ 60 anos	< 140
#5	2022	Saiz (Cochrane)	PICO diferente (intervenção e população)	7	7	HAS e DAC	135/85 ou menos
#6	2022	Ho	População de Idosos e tempo até benefício (PICO diferente)	2	9	≥ 65 anos	Não disponível
#7	2022	Deng	Não é uma Revisão Sistemática	0	0	Não disponível	Não disponível
#8	2021	Blood Pressure Lowering Treatment Trialists' Collaboration	Intevenção diferente (redução e não meta de PA)	4	48	Com e sem doença CV	categorias de 10mmHg
#9	2020	D'Anci	Revisão Sistemática Qualitativa	4	8	HAS	-10mmHg ou categorias de 10mmHg
#10	2020	García-Zamora	Qualidade baixa	4	4	HAS	<130/80
#11	2020	Baffour-Awuah	Intervenção (meta de tratamento intensivo) diferente	4	16	HAS	<150-160 ou <120-140

#12	2020	Arguedas (Cochrane)	Revisão atualizada no documento #5 Sainz				
#13	2019	Frey	Avalia efeitos colaterais e não tem meta-análise	6	15	HAS	variaram entre os estudos
#14	2019	Roush	Verifica se há diferença no controle da PA de acordo com a idade	3	16	HAS	120-140mmHg SBP (variado)
#15	2019	Takami	População de Idosos (PICO diferente)	3	6	>70 anos	<140
#16	2019	Sakima	PICO igual	3	19	HAS	<130/80 mmHg
#17	2019	Chi	Desfechos são só agrupados	2	4	HAS	variaram entre os estudos

DM = Diabetes Mellitus, HAS = Hipertensão Arterial Sistêmica, DAC = Doença Arterial Coronariana, PA = Pressão Arterial, PICO: população, intervenção, comparador, desfechos

Tabela 3S – referências das revisões sistemáticas listadas na tabela 2S.

#1	Seidu S, Willis H, Kunutsor SK, Khunti K. Intensive versus standard blood pressure control in older persons with or without diabetes: a systematic review and meta-analysis of randomised controlled trials. J R Soc Med. 2023 Apr;116(4):133-43. doi: 10.1177/01410768231156997. Epub 2023 Feb 24. PMID: 36825537; PMCID: PMC10164272.
#2	Yan Y, Zhang M, Wang X, Yang Q. Optimal blood pressure control target for older patients with hypertension: a systematic review and meta-analysis. Cardiovasc Innov Appl. 2023;7(1).
#3	Triatmaja R, Budhy T, Wulandari P. Intensive vs. standard blood pressure strategy in elderly patients: a systematic review and meta-analysis. J Hypertens. 2023;41(Suppl 2)
#4	Chen T, Ying A, Lin Z, Lu H. Time to clinical benefit of intensive blood pressure lowering in patients 60 years and older with hypertension: a secondary analysis of randomized clinical trials. JAMA Intern Med. 2022;182(6):660-7.
#5	Saiz LC, Arbeloa Á, Zabaleta-Del-Olmo E, et al. Blood pressure targets for the treatment of people with hypertension and cardiovascular disease. Cochrane Database Syst Rev. 2022;11.
#6	Ho VS, Huang J, Adams DH, et al. Time to benefit for stroke reduction after blood pressure treatment in older adults: a meta-analysis. J Am Geriatr Soc. 2022;70(5):1558-68.
#7	Não disponível
#8	Rahimi K, MacMahon S, Neal B, et al. Pharmacological blood pressure lowering for primary and secondary prevention of cardiovascular disease across different levels of blood pressure: an individual participant-level data meta-analysis. Lancet. 2021;397(10285):1625-36.
#9	D'Anci KE, Craven S, Leas B, et al. Effect of intensive blood pressure lowering on cardiovascular outcomes: a systematic review prepared for the 2020 US Department of Veterans Affairs/US Department of Defense Guidelines. Ann Intern Med. 2020;173(11):895-903.
#10	García-Zamora S, Zavaleta-Santos A, Álvarez-Bassols S, et al. Benefits and potential risks of intensive goals in the treatment of arterial hypertension–Systematic review and meta-analysis of clinical trials. Arch Cardiol Mex. 2020;90(4):444-52.
#11	Baffour-Awuah B, Dinberu MT, Andoh J, et al. Blood pressure control in older adults with hypertension: a systematic review with meta-analysis and meta-regression. Int J Cardiol Hypertens. 2020;6:100040.
#12	Arguedas, Jose Agustin, Viriam Leiva, and James M. Wright. "Blood pressure targets in adults with hypertension." *Cochrane Database of Systematic Reviews* 12 (2020).
#13	Frey L, Dürschmied D, Linnartz L, et al. Serious adverse events in patients with target-oriented blood pressure management: a systematic review. J Hypertens. 2019;37(11):2135-44.
#14	Roush GC, Sica DA, DiNicolantonio JJ, et al. Does the benefit from treating to lower blood pressure targets vary with age? A systematic review and meta-analysis. J Hypertens. 2019;37(8):1558-66.
#15	Takami Y, Kuriyama A, Yanagisawa H, et al. Target blood pressure level for the treatment of elderly hypertensive patients: a systematic review and meta-analysis of randomized trials. Hypertens Res. 2019;42(5):660-8.

#16	Sakima A, Kimura S, Tanaka T, et al. Optimal blood pressure targets for patients with hypertension: a systematic review and meta-analysis. Hypertens Res. 2019;42(4):483-95.
#17	Chi G, Huang W, Xiao D, et al. Effect of intensive versus standard blood pressure control on major adverse cardiac events and serious adverse events: a bivariate analysis of randomized controlled trials. Clin Exp Hypertens. 2019;41(2):160-7.

Tabela 4S - 1a busca por artigos originais.

**PUBMED**
(("Hypertension"[MeSH Terms] OR "Hypertension"[Title/Abstract] OR "blood pressure* high"[Title/Abstract] OR "high blood pressure*"[Title/Abstract]) AND ("intensive blood pressure"[Title/Abstract] OR "strict blood pressure"[Title/Abstract] OR "optimal blood pressure"[Title/Abstract] OR "blood pressure target"[Title/Abstract] OR "blood pressure goal"[Title/Abstract] OR "intensive"[Title/Abstract])) AND ((clinicaltrial[Filter] OR randomizedcontrolledtrial[Filter]) AND (2018:2024[pdat]))
**EMBASE**
#1 ('hypertension'/exp OR 'htn (hypertension)' OR 'acute hypertension' OR 'arterial hypertension' OR 'blood pressure, high' OR 'cardiovascular hypertension' OR 'controlled hypertension' OR 'endocrine hypertension' OR 'high blood pressure' OR 'high renin hypertension' OR 'hypertension' OR 'hypertensive disease' OR 'hypertensive effect' OR 'hypertensive reaction' OR 'hypertensive response' OR 'neurogenic hypertension' OR 'preexistent hypertension' OR 'salt high blood pressure' OR 'salt hypertension' OR 'secondary hypertension' OR 'systemic hypertension') AND ('antihypertensive agent'/exp OR 'anti hypertensic agent' OR 'anti hypertensive' OR 'anti hypertensive agent' OR 'anti hypertensive drug' OR 'antihypertension agent' OR 'antihypertensive' OR 'antihypertensive agent' OR 'antihypertensive agents' OR 'antihypertensive drug' OR 'antihypertensives' OR 'antihypertonic agent' OR 'hypotensiva' OR 'hypotensive' OR 'hypotensive agent' OR 'hypotensive drug') AND intensive AND ('randomized controlled trial'/exp OR 'controlled trial, randomized' OR 'randomised controlled study' OR 'randomised controlled trial' OR 'randomized controlled study' OR 'randomized controlled trial' OR 'trial, randomized controlled')<br>#2 #1 AND [embase]/lim NOT ([embase]/lim AND [medline]/lim) AND ([article]/lim OR [article in press]/lim OR [conference paper]/lim) AND [2018-2024]/py
**COCHRANE**
#1 MeSH descriptor: [Hypertension] explode all trees<br>#2 hypertension OR "high blood pressure" OR "blood pressure, high"<br>#3 #1 OR #2<br>#4 intensive OR "intensive blood pressure" OR "strict blood pressure" OR "optimal blood pressure" OR "blood pressure target" OR "blood pressure goal"<br>#5 #3 AND #4

Tabela 5S – características dos artigos originais

Principal Author	Year of Publication	Study Population Characteristics	BP Target in Intensive Group	Baseline Systolic BP (mmHg)	Baseline Diastolic BP (mmHg)	Total Number of Participants	Number of Events	Median Age	Percentage of Female Participants	Country of Origin	Missing Data n(%)
#01 BBB	1994	Hypertensive patients with well-controlled blood pressure	<80 mmHg (DBP)	155	95	2127	57	59.8	Not specified	Sweden	142 (6)
#02 Schrier	2002	Patients with normotensive type 2 diabetes	<130/80 mmHg	141.5	96	480	89	57	49%	USA	55 (11)
#03 Estacio	2006	Normotensive patients with type 2 diabetes and normo- or microalbuminu	<75mmHg (DBP)	126	84	129	5	56	47%	USA	10 (7)
#04 CardioSis	2009	Hypertensive patients with metabolic syndrome	<130 mmHg	144.0	92.0	1111	97	55.6	43%	Italy	27 (2%)
#05 Appel	2010	African American patients with hypertensive nephrosclerosis	<130/80 mmHg	142	95	1094	120	55.3	39%	USA	0 (0)
#06 ACCORD	2010	Patients with type 2 diabetes and hypertension	<120 mmHg	139.3	76.0	4733	460	62.2	38%	USA	232 (4,9)
#07 SPS3	2013	Patients with recent lacunar stroke	<130 mmHg	143.4	78.2	3020	377	63	37%	USA	550 (18,4)
#08 SPRINT	2015	Hypertensive patients at increased cardiovascular risk	<120 mmHg	139.7	78.1	9361	562	67.9	36%	USA	986 (10.5)
#09 HOMED-BP	2018	Japanese patients with hypertension > 40 years	<125/80	154	90	3518	51	59.6	50%	Japan	710 (20)
#10 Zhang	2021	Chinese patients aged 60-80 with hypertension	110-130 mmHg	146.1	86.1	8511	355	66.2	54%	China	234 (2,7)

Prinnjcipal Author	Year of Publication	Primary Outcomes	CVE in Intensive Group	CVE in Standard Group	Death in Intensive Group	Death in Standard Group	MI in Intensive Group	MI in Standard Group	Stroke in Intensive Group	Stroke in Standard Group	ROB 2.0
#01 BBB	1994	Level of blood pressure, side-effects, morbidity, and mortality	28/?	29/?	8/?	3/?	20/?	18/?	8/?	11/?	SC
#02 Schrier	2002	Change in in creatinine clearance	41/237	48/243	18/237	20/243	19/237	15/243	4/237	13/243	HIGH
#03 Estacio	2006	Change in urinary albumin excretion (UAE)	3/66	2/63	1/66	0/63	Not specified	Not specified	Not specified	Not specified	SC
#04 CardioSis	2009	Reduction in left ventricular mass index	12/557	20/553	4/557	5/553	4/557	6/553	4/557	9/553	HIGH
#05 Appel	2010	Composite of death, end-stage renal disease (ESRD), or decline in GFR	83/540	99/554	38/540	47/554	19/540	23/554	26/540	29/554	SC
#06 ACCORD	2010	Composite of nonfatal MI, nonfatal stroke, and cardiovascular death	310/2363	345/2371	150/2363	144/2371	126/2363	146/2371	34/2363	55/2371	LOW
#07 SPS3	2013	Recurrent stroke	267/1501	293/1519	106/1501	101/1519	36/1501	40/1519	125/1501	152/1519	SC
#08 SPRINT	2015	Composite of MI, ACS, stroke, heart failure, or cardiovascular death	314/4678	396/4683	155/4678	210/4683	97/4678	116/4683	62/4678	70/4683	LOW
#09 HOMED-BP	2018	Composite of cardiovascular death, myocardial infarction and stroke.	72/1759	75/1759	27/1759	31/1759	25/1759 (***)	28/1759 (***)	20/1759	16/1759	HIGH
#10 Zhang	2021	Composite of cardiovascular events and mortality	170/4243	217/4268	67/4243	64/4268	55/4243 (**)	82/4268 (**)	48/4243	71/4268	LOW

Principal Author	Year of Publication	Follow-Up	Progression to Stage 4/5 CRD in Intensive Group	Progression to Stage 4/5 CRD in Standard Group	Major Adverse Events in the Elderly Intensive Group	Major Adverse Events in the Elderly Standard Group	CVE in Diabetic Patients Intensive Group	CVE in Diabetic Patients Standard Group	SAE in Intensive Group	SAE in Intensive Group
#01 BBB	1994	4,9y	Not specified	Not specified	Not specified	Not specified	Not specified	Not specified	Not available	Not available
#02 Schrier	2002	5,3y	No difference	No difference	Not specified	Not specified	41/237	48/243	Not specified	Not specified
#03 Estacio	2006	1,9y	No difference	No difference	Not specified	Not specified	3/66	2/63	No difference	No difference
#04 CardioSis	2009	2	Not available	Not available	Not specified	Not specified	Not specified	Not specified	Not specified	Not specified
#05 Appel	2010	>= 3y	78/121	91/176	Not specified	Not specified	Not specified	Not specified	Not specified	Not specified
#06 ACCORD	2010	4.7 y	Not specified	Not specified	Not specified	Not specified	Not specified	Not specified	77/2363	30/2371
#07 SPS3	2013	3y	Not specified	Not specified	Not specified	Not specified	Not specified	Not specified	Not specified	Not specified
#08 SPRINT	2015	3.26y	14/1330*	15/1316*	640/1317($)	638/1319($)	Excluded	Excluded	220/4678	118/4683
#09 HOMED-BP	2018	5,3y	Not specified	Not specified	Not specified	Not specified	Not specified	Not specified	Not specified	Not specified
#10 Zhang	2021	3,34y	Not specified	Not specified	21/4243	21/4268	Not specified	Not specified	21/4243	21/4268

SAE = Serious Adverse Events / Stage 4 CRD GFR 15-29 ml/min/1,73 m^2 / Stage 5 CRD GFR < 15 ml/min/1,73 m^2

OBS: O estudo #01 BBB foi excluído por dados faltantes.

Tabela 6S – AMSTAR 2 das revisões sistemáticas adicionais utilizadas para recomendações clínicas adicionais.

AMSTAR 2	1	2	3	4	5	6	7	8	9	10	11	12	13	14	15	16	OVERALL
Seidu	Y	Y	Y	Y	Y	Y	N	Y	Y	N	Y	Y	Y	Y	Y	Y	Low
Trialists	Y	Y	Y	Y	Y	Y	Y	Y	Y	N	Y	Y	Y	Y	N	Y	Moderate

Tabela 7S - PRISMA da revisão sistemática.

Item	Descrição	Local
TÍTULO - 1	Identificar o relatório como uma revisão sistemática.	1
RESUMO - 2	Ver o checklist PRISMA 2020 para resumos.	2
INTRODUÇÃO - Racional - 3	Descrever a justificativa para a revisão no contexto do conhecimento existente.	3
INTRODUÇÃO - Objetivos - 4	Fornecer uma declaração explícita dos objetivos ou perguntas que a revisão aborda.	4
MÉTODOS - Critérios de Elegibilidade - 5	Especificar os critérios de inclusão e exclusão da revisão e como os estudos foram agrupados.	5
MÉTODOS - Fontes de Informação - 6	Especificar todas as bases de dados, registros, sites, organizações e outras fontes consultadas.	5
MÉTODOS - Estratégia de Busca - 7	Apresentar as estratégias de busca completas para todas as bases de dados e sites.	1S/4S
MÉTODOS - Processo de Seleção - 8	Especificar os métodos para decidir se um estudo atendia aos critérios de inclusão da revisão.	5
MÉTODOS - Processo de Coleta de Dados - 9	Especificar os métodos para coleta de dados dos relatórios, incluindo detalhes de ferramentas.	Texto Suplemento
MÉTODOS - Itens de Dados - 10a	Listar e definir todos os desfechos para os quais os dados foram procurados.	4
MÉTODOS - Outras Variáveis de Dados - 10b	Listar e definir todas as outras variáveis para as quais os dados foram procurados (ex: características dos participantes, fontes de financiamento).	Texto Suplemento
MÉTODOS - Avaliação de Risco de Viés - 11	Especificar os métodos para avaliar o risco de viés nos estudos incluídos.	6
MÉTODOS - Medidas de Efeito - 12	Especificar para cada desfecho as medidas de efeito usadas.	Fig 1/2 Tabela 3

MÉTODOS - Métodos de Síntese - 13a	Descrever os processos usados para decidir quais estudos eram elegíveis para cada síntese.	Texto Suplemento
MÉTODOS - Preparação de Dados - 13b	Descrever métodos necessários para preparar os dados para apresentação ou síntese.	Texto Suplemento
MÉTODOS - Exibição de Resultados - 13c	Descrever métodos para exibir visualmente os resultados dos estudos individuais.	Tabelas 1 e 5S
MÉTODOS - Métodos de Síntese - 13d	Descrever métodos usados para sintetizar resultados e fornecer justificativa para as escolhas.	Texto Suplemento
MÉTODOS - Heterogeneidade - 13e	Descrever métodos para explorar causas de heterogeneidade entre resultados dos estudos.	15
MÉTODOS - Análises de Sensibilidade - 13f	Descrever análises de sensibilidade conduzidas para avaliar a robustez dos resultados.	6
MÉTODOS - Avaliação de Viés de Relato - 14	Descrever métodos usados para avaliar o risco de viés devido a resultados ausentes.	Texto Suplemento
MÉTODOS - Avaliação de Certeza - 15	Descrever métodos usados para avaliar a certeza (ou confiança) no corpo de evidências.	5
RESULTADOS - Seleção de Estudos - 16a	Descrever os resultados do processo de busca e seleção dos estudos.	6
RESULTADOS - Estudos Excluídos - 16b	Citar estudos que atendiam aos critérios, mas foram excluídos, e justificar exclusões.	6
RESULTADOS - Características dos Estudos - 17	Citar cada estudo incluído e apresentar suas características.	6<br>Tabela 1
RESULTADOS - Risco de Viés nos Estudos - 18	Apresentar as avaliações de risco de viés para cada estudo incluído.	Tabelas 2
RESULTADOS - Resultados dos Estudos Individuais - 19	Apresentar, para cada estudo, as estatísticas resumidas e estimativas de efeito.	Tabela 1
RESULTADOS - Sínteses de Resultados - 20a	Resumir características e risco de viés entre estudos incluídos em cada síntese.	Tabela 1
RESULTADOS - Estatísticas de Síntese - 20b	Apresentar resultados de todas as sínteses estatísticas conduzidas.	9
RESULTADOS - Heterogeneidade - 20c	Apresentar resultados de investigações de causas de heterogeneidade.	15
RESULTADOS - Análises de Sensibilidade - 20d	Apresentar resultados de análises de sensibilidade conduzidas para avaliar robustez dos resultados.	6

RESULTADOS - Viés de Relato - 21	Apresentar avaliações de risco de viés devido a resultados ausentes para cada síntese.	Não disponível
RESULTADOS - Certeza das Evidências - 22	Apresentar avaliações de certeza no corpo de evidências para cada desfecho avaliado.	Tabela 3
DISCUSSÃO - Interpretação dos Resultados - 23a	Interpretar os resultados no contexto de outras evidências.	11
DISCUSSÃO - Limitações da Evidência - 23b	Discutir quaisquer limitações da evidência incluída na revisão.	15
DISCUSSÃO - Limitações da Revisão - 23c	Discutir quaisquer limitações dos processos de revisão usados.	15
DISCUSSÃO - Implicações - 23d	Discutir implicações dos resultados para prática, política e pesquisa futura.	1
OUTRAS INFORMAÇÕES - Registro e Protocolo - 24a	Fornecer informações de registro da revisão, incluindo nome e número de registro.	4
OUTRAS INFORMAÇÕES - Acesso ao Protocolo - 24b	Indicar onde o protocolo da revisão pode ser acessado.	4
OUTRAS INFORMAÇÕES - Emendas ao Protocolo - 24c	Descrever e explicar quaisquer emendas às informações fornecidas no protocolo.	Não disponível
OUTRAS INFORMAÇÕES - Suporte - 25	Descrever fontes de apoio financeiro ou não-financeiro para a revisão.	4
OUTRAS INFORMAÇÕES - Conflitos de Interesse - 26	Declarar quaisquer conflitos de interesse dos autores da revisão.	6
OUTRAS INFORMAÇÕES - Disponibilidade de Dados e Materiais - 27	Indicar quais materiais (dados, códigos) estão publicamente disponíveis e onde encontrá-los.	Texto Suplemento

Figura 1S – Metanálises dos desfechos individuais.

Desfecho: MORTE

Estudo	Intervenção Eventos	Intervenção Total	Controle Eventos	Controle Total	RR	IC 95%
Schrier	18	237	20	243	0.92	[0.50; 1.70]
Estacio	1	66	0	63	2.86	[0.12; 69.03]
CardioSis	4	557	5	553	0.79	[0.21; 2.94]
Appel	38	540	47	554	0.83	[0.55; 1.25]
ACCORD	150	2363	144	2371	1.05	[0.84; 1.30]
SPS3	106	1501	101	1591	1.11	[0.85; 1.45]
SPRINT	155	4678	210	4683	0.74	[0.60; 0.91]
HOMED-BP	27	1759	31	1759	0.87	[0.52; 1.45]
Zhang	67	4243	64	4268	1.05	[0.75; 1.48]
**Modelo de efeito randômico**		**15944**		**16085**	**0.93**	**[0.80; 1.09]**

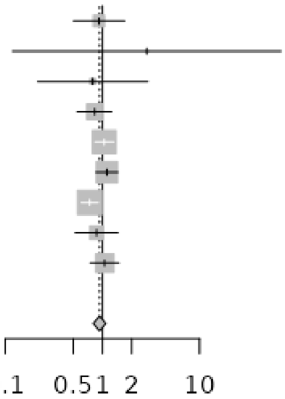

Heterogeneidade: $I^2 = 13\%$, $\tau^2 = 0.0149$, $p = 0.33$

Desfecho: IAM

Estudos	Intervenção Eventos	Intervenção Total	Controle Eventos	Controle Total	RR	IC 95%
Schrier	19	237	15	243	1.30	[0.68; 2.49]
CardioSis	4	557	6	553	0.66	[0.19; 2.33]
Appel	19	540	23	554	0.85	[0.47; 1.54]
ACCORD	126	2363	146	2371	0.87	[0.69; 1.09]
SPS3	36	1501	40	1591	0.95	[0.61; 1.49]
SPRINT	97	4678	116	4683	0.84	[0.64; 1.09]
HOMED-BP	25	1759	28	1759	0.89	[0.52; 1.52]
Zhang	55	4243	82	4268	0.67	[0.48; 0.95]
**Modelo de efeito randômico**		**15878**		**16022**	**0.85**	**[0.74; 0.97]**

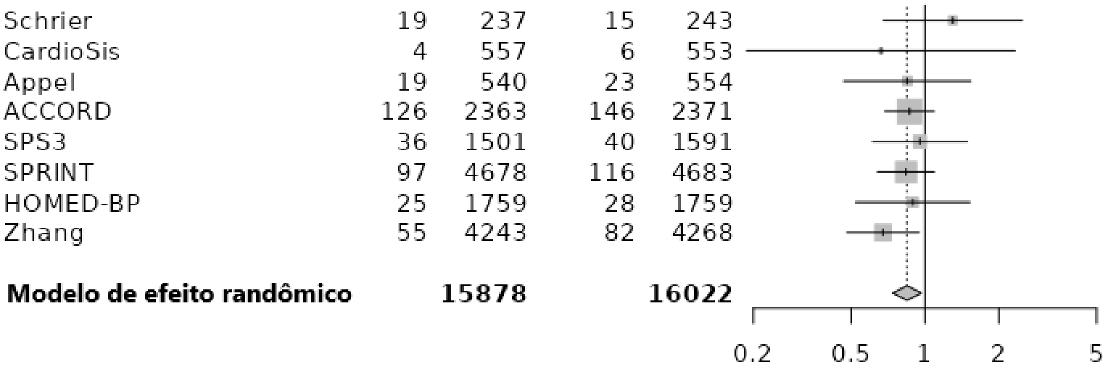

Heterogeneidade: $I^2 = 0\%$, $\tau^2 = 0$, $p = 0.79$

Desfecho: AVC

Estudo	Intervenção Eventos	Intervenção Total	Controle Eventos	Controle Total	RR	IC 95%
Schrier	4	237	13	243	0.32	[0.10; 0.95]
CardioSis	4	557	9	553	0.44	[0.14; 1.42]
Appel	26	540	29	554	0.92	[0.55; 1.54]
ACCORD	34	2363	55	2371	0.62	[0.41; 0.95]
SPS3	125	1501	152	1591	0.87	[0.70; 1.09]
SPRINT	62	4678	70	4683	0.89	[0.63; 1.24]
HOMED-BP	20	1759	16	1759	1.25	[0.65; 2.40]
Zhang	48	4243	71	4268	0.68	[0.47; 0.98]
**Modelo de efeito randômico**		**15878**		**16022**	**0.80**	**[0.70; 0.93]**

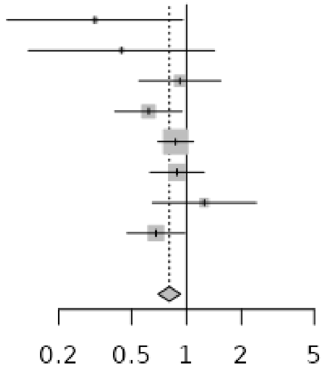

Heterogeneidade: $I^2 = 21\%$, $\tau^2 < 0.0001$, $p = 0.27$

OBS: O estudo Estacio não apresentou dados individualizados para IAM e AVC.

Figura 2S – Metanálise dos desfechos compostos sem o estudo SPRINT.

Estudo	Intervenção Eventos	Intervenção Total	Controle Eventos	Controle Total	Risco Relativo	RR	IC 95%
Schrier	41	237	48	243		0.88	[0.60; 1.28]
Estacio	3	66	2	63		1.43	[0.25; 8.29]
CardioSis	12	557	20	553		0.60	[0.29; 1.21]
Appel	83	540	99	554		0.86	[0.66; 1.12]
ACCORD	310	2363	345	2371		0.90	[0.78; 1.04]
SPS3	267	1501	293	1591		0.97	[0.83; 1.12]
HOMED-BP	72	1759	75	1759		0.96	[0.70; 1.32]
Zhang	170	4243	217	4268		0.79	[0.65; 0.96]
**Modelo de efeito randômico**		**11266**		**11402**		**0.89**	**[0.82; 0.97]**

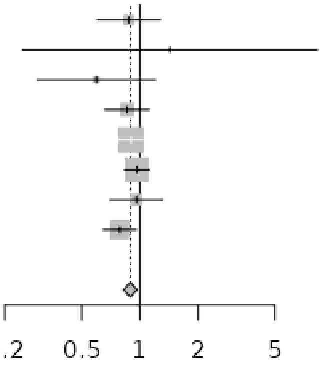

Heterogeneidade: $I^2 = 0\%$, $\tau^2 = 0$, $p = 0.73$

8. Metodologia detalhada

Critérios de Elegibilidade (MÉTODOS - 5)

Os artigos incluíram ensaios clínicos randomizados (ECRs) que compararam metas intensivas de controle da pressão arterial (<130/80 mmHg) com metas convencionais (≥130/80 mmHg) em adultos com 18 anos ou mais. Foram selecionados estudos com pelo menos um dos seguintes desfechos: mortalidade, infarto do miocárdio, acidente vascular cerebral, progressão para doença renal crônica (estágios IV ou V), necessidade de diálise ou transplante renal.

Fontes de Informação (MÉTODOS - 6)

As buscas foram conduzidas nas bases de dados Medline, Embase e Cochrane Library até maio de 2024. Foram realizadas duas buscas: a primeira focada em revisões sistemáticas (anos 2014 a 2024) e a segunda para ensaios clínicos randomizados. A estratégia de busca e outros dados foram armazenados como documentos em PDF ou Microsoft no armazenamento em nuvem OneDrive, compartilhado entre editores e metodologistas.

3. Estratégia de Busca (MÉTODOS - 7)

As estratégias de busca incluíram termos MeSH e palavras-chave relacionadas à hipertensão e controle intensivo da pressão arterial. Detalhes completos das estratégias estão nas tabelas 1S e 4S do material suplementar.

4. Processo de Seleção (MÉTODOS - 8)

O processo de seleção foi conduzido e armazenado no software Rayyan. Inicialmente, o recurso de detecção automática do Rayyan foi utilizado para identificar estudos duplicados, com um limite de 95% ou DOI (Digital Object Identifier). Dois revisores independentes participaram colaborativamente em cada etapa, avaliando inicialmente 10% dos artigos pelo título e resumo para garantir que um acordo seja alcançado. Esse procedimento de seleção em pares continuou até se atingir uma taxa de concordância de 90%, momento em que cada revisor avaliou um subconjunto de estudos ou um único revisor avaliaou os estudos restantes. Divergências foram resolvidas por consenso. A seleção de textos completos por textos completos foi realizada coma avaliação de todos os documentos pelos dois pesquisadores de forma independente.

5. Processo de Coleta de Dados (MÉTODOS - 9)

A coleta de dados foi realizada usando tabela específica para extração pré-determinada, incluindo as características dos estudos, desfechos e qualidade metodológica. Um metodologista extraiu as características basais e dados de desfecho dos estudos com ajuda desoftware de inteligência artificial (ChatGPT). Em seguida, um segundo revisor extraiu os dados dos desfechos e verificou todos os outros dados relacionados à publicação para garantir precisão, integridade e confiabilidade dos achados. Os dados dos desfechos extraídos pelos dois revisores foram comparados e, em caso de discrepâncias, foram avaliados conjuntamente até que um consenso seja alcançado. Esse processo em duas etapas assegura um alto nível de precisão e confiança nas informações apresentadas.

6. Itens de Dados (MÉTODOS - 10a)

Os principais desfechos incluíram mortalidade, infarto do miocárdio, acidente vascular cerebral e progressão para doença renal avançada. Dados adicionais foram coletados para eventos adversos graves, como hipotensão e lesão renal aguda.

7. Outras Variáveis de Dados (MÉTODOS - 10b)

Outras variáveis incluíram: autor principal, ano de publicação, população do estudo, incluindo suas características, tais como: idade mediana dos participantes, percentual de participantes do sexo feminino, descrição da intervenção e do comparador utilizado, país de origem do estudo, fontes de financiamento, número total de participantes e eventos.

8. Avaliação de Risco de Viés (MÉTODOS - 11)

A avaliação de risco de viés foi realizada com a ferramenta Risk of Bias 2 (RoB 2) da Colaboração Cochrane para os ECRs, aplicada por dois revisores independentes em todos os estudos incluídos.

9. Medidas de Efeito (MÉTODOS - 12)

As medidas de efeito utilizadas incluíram risco relativo para cada desfecho, como mortalidade e eventos cardiovasculares, com intervalos de confiança de 95%.

10. Métodos de Síntese (MÉTODOS - 13a)

A síntese dos dados foi conduzida por meta-análise através de aplicativo específico, que pode ser acessado pelo site específico (http://shiny.nbbinc.info/metaECR).

11. Preparação de Dados (MÉTODOS - 13b)

Os dados foram preparados para síntese e exibição em tabelas e figuras detalhadas no artigo principal e no material suplementar, seguindo o padrão GRADE e PRISMA para visualização dos resultados, certeza da evidência e dos processos de inclusão e exclusão.

12. Exibição de Resultados (MÉTODOS - 13c)

Os resultados foram exibidos em tabelas e figuras (como na Figura 1S), detalhando os desfechos primários e secundários com visualizações de meta-análise.

13. Métodos de Síntese (MÉTODOS - 13d)

A meta-análise foi conduzida usando modelos de efeito randômico.

14. Heterogeneidade (MÉTODOS - 13e)

A heterogeneidade foi explorada utilizando o índice I^2, teste Chi quadrado e avaliação das medidas de efeito no gráfico Forest Plot.

15. Análises de Sensibilidade (MÉTODOS - 13f)

Análises de sensibilidade foram conduzidas excluindo estudos com moderado e alto risco de viés para avaliar a robustez dos resultados.

16. Avaliação de Viés de Publicação (MÉTODOS - 14)

O viés de publicação não foi avaliado pela razão do número de estudos originais ser menor do que dez.

17. Avaliação de Certeza (MÉTODOS - 15)

A certeza das evidências foi avaliada usando a metodologia GRADE, considerando a qualidade, consistência, a precisão, a presença de evidência indireta relativa à pergunta PICO deste documento, e analisando o viés de publicação.

Esses métodos foram seguidos para garantir rigor e transparência na avaliação dos efeitos de metas intensivas de pressão arterial, baseando-se nas melhores práticas de revisão sistemática e meta-análise.
