# Supplementary material for: Revisão Sistemática sobre a Eficácia de Metas Intensivas do Tratamento Anti-Hipertensivo: Recomendação da Sociedade Brasileira de Cardiologia (SBC)
Source: Arq Bras Cardiol. 2025 Mar 18;122(3):e20240761. [Article in Portuguese] doi: 10.36660/abc.20240761 (PMC12013737; doi:10.36660/abc.20240761)
Supplement: Translation

# Translation: Systematic Review of the Effectiveness of Intensive Antihypertensive Treatment Goals: Brazilian Society of Cardiology (SBC) Recommendation

**DOI:** 10.36660/abc.20240761i

**Authors:** Andréa Araujo Brandão, Cibele Isaac Saad Rodrigues, Luiz Aparecido Bortolotto, Leonardo Castro Luna, Bruno Monteiro Barros, Mario Fritsch Toros Neves, Ana Flávia de Souza Moura, Frida Liane Plavnik, Luciano Ferreira Drager, Osni Moreira, Weimar Kunz Sebba Barroso de Souza, Wilson Nadruz

**Affiliations:** 1 Brazil Faculdade de Ciências Médicas – Universidade do Estado do Rio de Janeiro, Rio de Janeiro, RJ – Brazil; 2 Brazil Pontifícia Universidade Católica de São Paulo, Sorocaba, SP – Brazil; 3 Brazil Instituto do Coração do Hospital das Clínicas da Faculdade de Medicina da Universidade de São Paulo, São Paulo, SP – Brazil; 4 Brazil Instituto Nacional de Cardiologia, Rio de Janeiro, RJ – Brazil; 5 Brazil Escola Bahiana de Medicina e Saúde Pública, Salvador, BA – Brazil; 6 Brazil Hospital Alemão Oswaldo Cruz, São Paulo, SP – Brazil; 7 Brazil Clínica Gapski Moreira, Curitiba, PR – Brazil; 8 Brazil Universidade Federal de Goiás, Goiânia, GO – Brazil; 9 Brasil Faculdade de Ciências Médicas, Universidade Estadual de Campinas (UNICAMP), Campinas, SP – Brazil

**Keywords:** Hypertension, Systematic Review, Meta-Analysis, Drug Therapy

## Abstract

**Background:**

Strict blood pressure control has been investigated as a strategy to reduce severe cardiovascular events in patients with hypertension. However, there are still doubts about the impact of intensive antihypertensive treatment goals (< 130/80 mmHg) compared to conventional goals (≥ 130/80 mmHg) in preventing myocardial infarction, stroke, mortality, and possible treatment-related adverse effects.

**Objective:**

To evaluate the effectiveness of intensive antihypertensive treatment goals in reducing critical cardiovascular events compared to usual goals.

**Methods:**

This systematic review included randomized controlled trials (RCTs) that compared intensive blood pressure control goals with conventional goals in adults aged 18 years or older. Studies with at least one of the following outcomes were included: mortality, myocardial infarction, stroke, progression to stage 4 or 5 chronic kidney disease, need for dialysis, or kidney transplantation. Medline, Embase, and Cochrane Library databases were searched up to May 2024. Risk of bias assessment was performed by two independent reviewers using the Cochrane Collaboration’s Risk of Bias 2 (RoB 2) tool. Synthesis of results was conducted through meta-analysis for the composite outcome of myocardial infarction, stroke, and all-cause mortality. The certainty of scientific evidence and strength of recommendation followed the methods proposed by the Grading of Recommendations Assessment, Development, and Evaluation (GRADE) tool.

**Results:**

Nine RCTs with more than 34,000 participants were included. Intensive treatment was associated with a 13% reduction in cardiovascular events. In the studies with low risk of bias, the reduction was 17%, with high certainty of evidence. Separately, a significant reduction was observed in the outcomes of myocardial infarction and stroke, but not in all-cause mortality. Limited data were found on the progression of kidney disease and the need for dialysis or kidney transplantation.

**Conclusion:**

High-quality evidence suggests that more intensive antihypertensive treatment goals significantly reduce cardiovascular events. However, the choice of treatment goals should be individualized, considering factors such as age, frailty, individual cardiovascular risk, and the possibility of adverse events. Adherence to treatment is essential to therapeutic success.

**Recommendation 1:** The Brazilian Society of Cardiology recommends the strategy of more intensive antihypertensive treatment goals (values below 130/80 mmHg) for adult patients with hypertension, with the aim of reducing major cardiovascular events (myocardial infarction, stroke, and death). This is a strong recommendation with high certainty of evidence.

**Recommendation 2:** The Brazilian Society of Cardiology recommends the strategy of more intensive antihypertensive treatment goals (values below 130/80 mmHg) for elderly patients with hypertension (age > 65 years), with the aim of reducing future major cardiovascular events (myocardial infarction, stroke, and death). This is a strong recommendation with high certainty of evidence. This recommendation should be assessed on an individual basis when treating frail elderly patients or patients with limited life expectancy.

**Recommendation 3:** The Brazilian Society of Cardiology recommends the strategy of more intensive antihypertensive treatment goals (additional reduction of 5 mmHg) for patients with hypertension at high cardiovascular risk who are already within the intensive treatment range (SBP < 130 mmHg), with the objective of further reducing future major cardiovascular events (myocardial infarction, stroke, and death). This is a strong recommendation with high certainty of evidence.

## Introduction

Hypertension is currently is currently a major health challenge that substantially contributes to cardiovascular disease rates worldwide. It is a major risk factor for morbidity and mortality, affecting more than 1 billion people and accounting for approximately 9.4 million deaths annually.^1^ Not only does hypertension increase the risk of cardiovascular complications, it also imposes a considerable economic burden on health systems, especially in low- and middle-income countries.^2^ In Brazil, hypertension is also a major public health problem, affecting approximately one third of the adult population.^3^ It is responsible for a significant proportion of cases of myocardial infarction and stroke, which are the leading causes of death in Brazil.^4,5^ Hypertension also exerts a substantial influence on the Brazilian Unified Health System (SUS, acronym in Portuguese), resulting in increased health costs, significant loss of work days, and early retirement.^6^ There is a growing need for effective public health interventions aimed at prevention, early detection, and management of the disease.^7^ It is crucial to address this challenge in order to improve health and reduce chronic disease levels in Brazil.

Antihypertensive treatment plays a crucial role in disease management and in reducing the risk associated with cardiovascular events, usually involving a combination of lifestyle interventions and pharmacological treatments.^7^ There is currently a debate regarding the optimal goals to be achieved in hypertension. Intensive control strategies, which aim for lower blood pressure (BP) goals, have shown a significant reduction in the incidence of critical events in high-risk patients in clinical trials such as SPRINT.^8^ Nonetheless, this approach also involves a potential increase in the risk of adverse events, such as hypotension, acute kidney injury, and electrolyte imbalances, which can lead to unfavorable outcomes, especially in elderly or frail patients.^9^ On the other hand, conventional treatment, which follows more moderate BP goals, may not provide the same level of cardiovascular and renal protection.^8^This discussion highlights the importance of individualized treatments, which consider patients’ general health status, in addition to comorbidities and the possibility of treatment-related adverse effects.

With the objective of clarifying the best strategy in relation to BP goals in patients with hypertension, considering the best current scientific evidence, the Brazilian Society of Cardiology (SBC) has developed a clinical recommendation on this topic.

## Methods

In order to develop the clinical recommendation, a systematic review was carried out. The following research question was structured in the PICO (patient/population, intervention, comparison, and outcome) format: In patients with hypertension, what is the difference between pharmacological treatment with more intensive BP control goals (< 130/80 mmHg) compared to the usual control goals (≥ 130/80 mmHg), specifically in relation to critical and important outcomes, such as death, stroke, myocardial infarction, progression to stage 4 or 5 kidney failure, need for dialysis, or kidney transplant? The study protocol was registered in the PROSPERO database, under number CRD42024545853.

This document used rapid systematic review methodology, which belongs to the family of systematic reviews. This tool was developed over the last decade with the aim of maintaining methodological rigor in seeking the best possible evidence, but with modifications that speed up the execution time. These reviews generally inform medical societies or health institutions regarding the best evidence available, based on a question in the PICO format, in a sensitive, transparent, and systematic manner. Leading institutions in the area of methodology have described the methods for this type of systematic review.^10-12^

An initial search for systematic reviews on the PICO question was carried out in three databases: Medline, Embase, and Cochrane Library. Details of the search strategies are provided in the supplementary material (Table S1). Two researchers independently performed study selection and extraction of baseline characteristics from the documents selected for full reading (Tables S2 and S3). However, among the systematic reviews found, which covered the years 2019 to 2024, none exactly addressed the PICO question of interest, except for the paper by Sakima et al.^13^ Their publication included searches for studies up to March 2018, retrieving 9 articles that compared the intensive BP goal (< 130/80 mmHg) with the usual goal and evaluated cardiovascular events as an outcome. The authors decided to update the search for this systematic review and perform a new meta-analysis with the data extracted from the primary studies.

A second search for randomized controlled trials (RCTs) was performed to update the selected reference document, covering the period March 2018 to May 2024 in the same databases as the first search. Details of the search strategies are provided in the supplementary material (Table 4S). Articles selected for full-text review were considered for inclusion if they met the following criteria: (1) being RCTs; (2) presenting risk estimates to assess the impact of intensive BP goals versus usual BP goals; (3) presenting at least one of the following outcomes, described numerically: all-cause mortality, stroke, myocardial infarction, progression to stage 4 or 5 renal failure, need for dialysis, or kidney transplantation; (4) including patients aged 18 years or older. There were no language restrictions on study selection.

Two researchers were responsible for the selection, extraction, and quality assessment of all final articles. The Cochrane Collaboration’s Risk of Bias 2 (RoB 2) tool was used to assess the risk of bias.^14^ The strength of recommendations and the certainty of evidence were assessed following the Grading of Recommendations Assessment, Development, and Evaluation (GRADE) methodology^15^ (Tables 2 and 3). A detailed description of the methodology applied in this systematic review can be found in the supplementary material. Publication bias was not assessed because there were fewer than 10 original studies.

**Table 2:** – Risk of bias in the primary studies according to the Cochrane Collaboration’s RoB 2.0 tool

Study	Intervention	Comparator	Outcome	D1	D2	D3	D4	D5	
Schrier	Intensive treatment	Usual treatment	CV events						
ESTACIO	Intensive treatment	Usual treatment	CV events						
CardioSis	Intensive treatment	Usual treatment	CV events						
Appel	Intensive treatment	Usual treatment	CV events						
ACCORD	Intensive treatment	Usual treatment	CV events						
SPS3	Intensive treatment	Usual treatment	CV events						
SPRINT	Intensive treatment	Usual treatment	CV events						
HOMED-BP	Intensive treatment	Usual treatment	CV events						
STEP - Zhang	Intensive treatment	Usual treatment	CV events						

: Low risk; : Moderate risk; : High risk. D1: Randomization; D2: Deviation from treatment; D3: Missing data; D4: Outcome measurement; D5 Selection of reported results. CV: cardiovascular.

**Table 3:** – Summary of findings, according to GRADE methodology

Outcome	Absolute effect (95% CI)		Relative effect (95% CI)	Number of participants (studies)	Certainty of evidence
	Risk with usual treatment	Risk with intensive treatment (< 130/80 mmHg)			
CV events (death, AMI, and stroke)	89 per 1000	77 per 1000	RR 0.87 (0.80 to 0.94)	32,749 (9 RCTs)	High
Death	39 per 1000	36 per 1000	RR 0.93 (0.80 to 1.09)	32,029 (9 RCTs)	High

AMI: acute myocardial infarction; CV: cardiovascular; RCTs: randomized controlled trials.

The clinical recommendations were defined by means of consensus during a reunion of a recommendation panel made up of professionals appointed by the SBC. A threshold of 5% was considered as a minimum important difference for the intervention to be considered clinically relevant.

The project was supervised and funded in its entirety by the SBC. The preparation of the systematic review was conducted by an independent team of methodologists.

## Results

The 9 articles included in the reference meta-analysis,^13^ comparing the intensive antihypertensive treatment goal (< 130/80 mmHg) with the standard treatment goal, were assessed for inclusion in this new meta-analysis. One of them was excluded because it did not present the relative risks of interest,^16^ and the remaining 8 publications were included.^8,17-23^

As a result of the second literature search, with the objective of identifying new RCTs published after March 2018, 2,061 citations were initially screened. Only 2 of these studies were selected for full-text review, resulting in the inclusion of an additional RCT in the updated meta-analysis^24^ and the exclusion of the second article because it was not an original RCT.^25^

The main characteristics of the 9 primary studies included in this meta-analysis are displayed in Table 1. All extracted characteristics are available in the supplementary material (Table 5S).

**Table 1:** – Main characteristics of the primary studies

Main author	Year of publication	Population characteristics	BP goal in intensive group	Baseline systolic BP (mmHg)	Baseline diastolic BP (mmHg)	Number of participants	Events	Mean age	Percentage women	Country	Missing data. n(%)
#02 Schrier	2002	Normotensive patients with type 2 diabetes	<130/80 mmHg	141.5	96	480	89	57	49%	USA	55(11)
#3 Estacio	2006	Normotensive patients with type 2 diabetes and normo- or microalbuminuria	< 75 mmHg (PAD)	126	84	129	5	56	47%	USA	10(7)
#4 CardioSis	2009	Hypertensive patients with metabolic syndrome	<130 mmHg	144.0	92.0	1111	97	55.6	43%	Italy	27(2)
#5 Appel	2010	African American patients with hypertensive nephrosclerosis	<130/80 mmHg	142	95	1094	120	55.3	39%	USA	0(0)
#6 ACCORD	2010	Patients with type 2 diabetes and hypertension	<120 mmHg	139.3	76.0	4733	460	62.2	38%	USA	232(4.9)
#7 SPS3	2013	Patients with recent lacunar stroke	<130 mmHg	143.4	78.2	3020	377	63	37%	USA	550(18.4)
#8 SPRINT	2015	Hypertensive patients with increased cardiovascular risk	<120 mmHg	139.7	78.1	9361	562	67.9	36%	USA	986(10.5)
#9 HOMED-BP	2018	Japanese patients with hypertension over 40 years of age	<125/80	154	90	3518	51	59.6	50%	Japan	710(20)
#10 Zhang	2021	Chinese patients with hypertension between 60 and 80 years of age	110-130 mmHg	146.1	86.1	8511	355	66.2	54%	China	234(2.7)
**Main author**	**Year of publication**	**Primary outcomes**	**CV events, intensive group**	**CV events, usual group**	**Death, intensive group**	**Death, usual group**	**AMI, intensive group**	**AMI, usual group**	**Stroke, intensive group**	**Stroke, usual group**	**RoB 2.0**
#02 Schrier	2002	Altered creatinine clearance	41/237	48/243	18/237	20/243	19/237	15/243	4/237	13/243	
#3 Estacio	2006	Altered urinary albumin excretion	3/66	2/63	1/66	0/63	Not reported	Not reported	Not reported	Not reported	
#4 CardioSis	2009	Reduced left ventricular mass index	12/557	20/553	4/557	5/553	4/557	6/553	4/557	9/553	
#5 Appel	2010	Composite outcome of death, end-stage renal disease, or reduced glomerular filtration rate	83/540	99/554	38/540	47/554	19/540	23/554	26/540	29/554	
#6 ACCORD	2010	Composite outcome of AMI, stroke, and death	310/2363	345/2371	150/2363	144/2371	126/2363	146/2371	34/2363	55/2371	
#7 SPS3	2013	Recurrent stroke	267/1501	293/1519	106/1501	101/1519	36/1501	40/1519	125/1501	152/1519	
#8 SPRINT	2015	Composite outcome of AMI, stroke, cardiovascular death, ACS, and HF	314/4678	396/4683	155/4678	210/4683	97/4678	116/4683	62/4678	70/4683	
#9 HOMED-BP	2018	Composite outcome of cardiovascular death, AMI, and stroke	72/1759	75/1759	27/1759	31/1759	25/1759 (***)	28/1759 (***)	20/1759	16/1759	
#10 Zhang	2021	Composite outcome of cardiovascular events and death	170/4243	217/4268	67/4243	64/4268	55/4243 (**)	82/4268 (**)	48/4243	71/4268	

(**) ACS, not isolated AMI. (***) Not only AMI, ischemic heart disease included death from angina pectoris, cardiac arrest, and nonfatal myocardial infarction. ACS: acute coronary syndrome; AMI: acute myocardial infarction; BP: blood pressure; CV: cardiovascular; HF: heart failure

The effect for the primary composite outcome, obtained by summarizing the data from the 9 primary studies, is shown in Figure 1. There was a 13% reduction in events, favoring the strategy of more intensive antihypertensive treatment goals. For the same outcome, when only studies with low risk of bias were included (Figure 2), a 17% reduction in events was observed, also favoring the strategy of more intensive antihypertensive treatment goals. The certainty of evidence for this analysis was classified as high, according to the GRADE methodology.

**Figure 1:** – Meta-analysis for the primary composite outcome, defined as myocardial infarction, stroke, and all-cause mortality.

Heterogeneity: I^2^= 0%; t^2^ = 0.0013; p = 0.60

**Figure 2:** – Meta-analysis of the primary composite outcome, defined as myocardial infarction, stroke, and all-cause mortality, assessed only for studies with low risk of bias.

Heterogeneity: I^2^= 0%; t^2^ = 0.0005; p = 0.38

When these 3 outcomes were assessed separately, they showed a reduction in myocardial infarction and stroke, favoring the strategy of more intensive goals, with no significant difference observed for all-cause mortality (Figure 1S).

The outcome of progression to kidney disease was assessed in 4 of the included studies, but only 2 provided numerical data. Due to the low number of studies and differences in the definitions of kidney progression, the authors did not include this outcome in the primary assessment and did not perform a meta-analysis for this individual event.

Two additional systematic reviews assessed for full reading in the first search answered PICO questions with slight differences in relation to this review’s original scientific question. They were considered highly relevant to the health system by the authors, and they supported two additional clinical recommendations in this document.^26,27^ Both publications were considered to have good technical quality by the authors, and the AMSTAR 2 quality assessment^28^ penalized them only in relation to characteristics that did not compromise central information. Table 6S displays the AMSTAR 2 quality assessment of both additional systematic reviews used for supplemental clinical recommendations.

Table 7S describes this document’s PRISMA.

## Discussion

Hypertension is one of the main modifiable risk factors for premature death. Therefore, it is of paramount importance to determine whether more intensive BP reduction goals offer additional benefits for reducing cardiovascular complications, in line with the educational mission of the SBC. This review offers a conclusive answer to this gap in clinical practice.

Defining a target value for antihypertensive treatment has been a challenge in the scientific literature due to the variability of the goals proposed in different studies. Although a cutoff point of 120/80 mmHg can be considered, RCTs that aimed at this more rigorous target often faced difficulties in achieving these results. For this reason, this systematic review opted to include studies in which the intervention arm aimed at a BP below 130/80 mmHg, a more achievable objective in real clinical practice.

In the initial literature search, with the objective of identifying systematic reviews that addressed the predefined PICO question, no document was found that could provide an updated answer. This led to the decision to update the article by Sakima et al.^14^

Based on the results demonstrated in this review, which included data from more than 34,000 individuals, the clinical benefit of stricter BP control goals for the population of people with hypertension has been well established. However, there was still uncertainty as to whether all cardiovascular risk spectra would benefit from this effect. Among the 9 studies included, 5 of them (with more than 6,000 patients) had a mean age below 60 years and a significant proportion of women (40% or more). This allows us to conclude that a substantial proportion of these participants were in the low-risk category for cardiovascular events (estimated risk < 7.5% over 10 years). On the other hand, the remaining 4 studies, which included mostly male patients over 60 years, adequately represented the other extreme end of exposure, encompassing patients with high cardiovascular risk (estimated risk > 15% over 10 years). Combining these data, we can assume that these subgroups were well represented in the studies that observed an important benefit in event reduction.

Another relevant question that arises is whether further reductions to values of 120/80 mmHg or even lower, particularly in patients with higher cardiovascular risk and lower likelihood of side effects, could result in additional clinical benefits (and would be worth recommending, if put into practice). Based on individual data meta-analysis from the 2021 Blood Pressure Lowering Treatment Trialists’ Collaboration, for any initial BP range (even in individuals with systolic values below 120 mmHg), a reduction of 5 mmHg leads to an 11% reduction in cardiovascular events.^28^ For these high-risk individuals, pursuing the goals of strict control proposed by the SPRINT^8^ and ACCORD^22^ studies may be an advantageous therapeutic option. The risk of side effects, number of medications used (adherence), and additional costs must be taken into account and compared with the significant reduction in critical cardiovascular events. This systematic review served as a reference for the third clinical recommendation in this document.

Stricter BP control goals always raise concerns about adverse events, especially in very elderly or frail patients. However, a recent meta-analysis involving 20,895 elderly individuals demonstrated that a strategy of intensive BP control goals resulted in a 29% reduction in the main cardiovascular events, with no evidence of an increase in serious adverse reactions or worsening of renal function.^27^ These findings further support the implementation of stricter hypertension control for the general population, regardless of age. Nonetheless, this recommendation does not eliminate the need to consider the individual circumstances of each patient and to establish less intensive goals for patients who are frail, have a limited life expectancy, or possess characteristics that may increase the risk of adverse effects. This systematic review served as a reference for the second clinical recommendation in this document.

Regarding possible adverse events of antihypertensive treatment, there is also discussion about the J-curve phenomenon. This refers to the observation that both excessively high and excessively low BP levels are associated with an increased risk of cardiovascular complications, forming a J-shaped curve when plotted on a graph. This phenomenon is particularly observed with diastolic BP and its association with coronary artery disease and other cardiovascular events. The concept of the J-curve relationship between BP and cardiovascular outcomes was first proposed in the 1970s,^30^ and it was subsequently verified in several observational studies, such as the Framingham Study.^31^ Based on the same systematic review carried out by the Blood Pressure Lowering Treatment Trialists’ Collaboration group in 2021, bringing together 48 RCTs (which have more robust certainty of evidence than observational studies), when we evaluated the population with previous cardiovascular disease and reductions in systolic BP beyond 120 mmHg, no increase was shown in cardiovascular events. On the contrary, even in this population with very high risk, a decrease in primary outcomes was observed with very intensive BP reduction goals, contradicting the hypothesis of the J-curve phenomenon.

An additional relevant discussion is whether the method of measuring BP could influence the clinical effect results observed. The SPRINT study used a method that involved taking 3 BP measurements during an office visit, with the patient seated, after a 5-minute rest period, using an automated measurement system (Model 907, Omron Healthcare), without the presence of a physician or other health care professional. This procedure has been criticized for differing from those used in other studies, raising concerns about the reproducibility of its results. However, a study carried out shortly after SPRINT demonstrated, through interviews with its participants, that this technique was implemented in less than half of the patients, with no differences in clinical outcomes between the groups based on the BP measurement method.^32^ A second study compared the technique proposed in the SPRINT trial with BP measurements taken without prior rest. The results showed considerable variability in the agreement between values, suggesting that the SPRINT technique could lead to lower BP readings.^33^ However, it was an observational study, conducted in a single center, with a small number of participants. These critiques are important, but they do not invalidate the results presented in this meta-analysis. Thus, one of the many advantages of conducting a systematic review is that it summarizes the results of several studies, minimizing the influence of a single isolated article on the final result. When performing sensitivity analysis excluding the SPRINT study, the clinical benefit of intensive BP reduction remained, showing an 11% reduction in events (Figure 2S).

A recent systematic review in the process of being published assessed a similar PICO question and reached similar results, corroborating the clinical recommendations suggested in this document by the SBC.^34^ Whelton et al. (2024) observed a 22% reduction for the intensive systolic BP control goal (< 130 mmHg) for a composite outcome of stroke, coronary heart disease, heart failure, and cardiovascular death (hazard ratio [HR] = 0.78; 95% confidence interval [CI] = 0.70 to 0.87; heterogeneity: I^2^ = 64.5%; p = 0.01). There was a statistical difference for the outcome of overall mortality (HR = 0.89; 95% CI = 0.79 to 0.99; heterogeneity: I^2^ = 37%; p = 0.14), although there was some degree of imprecision for the clinical importance of this effect, as shown by the CI.

Finally, an RCT that was published while this paper was in the final composition phase also corroborates this systematic review’s findings.^35^ Liu et al. included 11,255 Chinese participants with high cardiovascular risk (4,359 with diabetes and 3,022 with prior stroke) who were assigned to intensive treatment (n = 5,624) or standard treatment (n = 5,631). The mean age was 64.6 years. The mean systolic BP during follow-up was 119.1 mmHg (standard deviation: 11.1) in the intensive treatment group and 134.8 mmHg (standard deviation: 10.5) in the standard treatment group. During a median of 3.4 years of follow-up, primary outcomes occurred in 547 (9.7%) participants in the intensive treatment group and 623 (11.1%) in the standard care group (HR = 0.88; 95% CI = 0.78 to 0.99). Severe adverse events of syncope were infrequent and occurred more frequently in the intensive treatment group (n = 24 [0.4%] of 5,624) than in the standard care group (n = 8 [0.1%] of 5,631; HR = 3.00, 95% CI = 1.35 to 6.68). There were no significant differences between groups regarding the severe adverse events of hypotension, electrolyte abnormality, injurious fall, or acute kidney injury.

Other factors, in addition to clinical benefits and harms, should be considered by a recommendation panel when making the final decision for a clinical recommendation, according to the GRADE Evidence-to-Decision framework. Cost is one of these factors. In Brazil, the main classes of antihypertensive medications are provided free of charge throughout the country by the Ministry of Health, which facilitates the implementation of the intensive strategy recommended in this document.^36^

Patient adherence to prescribed medications can be challenging, as stricter BP control goals usually require increased medication use to achieve desired results. Strategies to improve adherence, such as simplifying dosing regimens (prescribing medications that require fewer doses per day),^37^ using single-pill combinations,^38^ encouraging patients to monitor their BP at home,^39^ and fostering a close relationship between healthcare professionals and patients,^40^ can contribute to the success of this strategy.

Another approach to improving patient adherence is to involve patients in the decision-making process. Using cardiovascular risk prediction calculators, it is possible to determine the individual probability of cardiovascular events over the next 10 years.^41^ Based on this estimate, patients can be informed about the real meaning of an additional BP reduction and, consequently, future cardiovascular risk, also considering the possible disadvantages (adverse events, cost, adherence, etc.). This promotes a better understanding of the proposed strategy as a whole, as well as the strategy’s permanent effects.

There are some limitations to this systematic review and meta-analysis. First, there is an understandable heterogeneity between the characteristics of the various studies on the topic. They differ in several aspects, including the definitions of intensive antihypertensive treatment goals, participants’ mean age, BP measurement methods, and the baseline cardiovascular risk of the patients included, among others. Second, the studies have limited information regarding the progression of kidney disease in patients already affected by chronic kidney disease. Although this was a predefined clinical outcome in the protocol, it was not widely investigated in the included studies, which prevents the formulation of definitive conclusions. Finally, the follow-up time of the studies varied between 2 and 5 years, approximately. The treatment time for hypertension is undefined, from the outset, for the total remaining life of the patient with hypertension. Therefore, the clinical effect demonstrated in a limited number of years may be underestimated for a much longer-term clinical strategy.

## Conclusion

Hypertension continues to pose an immense health challenge, with profound implications for cardiovascular morbidity and mortality in the Brazilian population. The findings of this review highlight the benefits of intensive antihypertensive treatment goals, which have been shown to significantly reduce the incidence of major cardiovascular events. However, the decision to adopt more aggressive BP control goals should be individualized, taking into account patient-specific factors such as age, frailty, and risk of adverse events. Moreover, ensuring patient adherence to prescribed medication regimens is crucial to the success of any treatment strategy. By considering potential risks and benefits and involving patients in the final decision-making process, health professionals can optimize the management of hypertension and improve clinical outcomes for patients and health systems.
